# Porous Organic Cages as Building Blocks for Framework Materials

**DOI:** 10.1002/anie.202509618

**Published:** 2025-08-01

**Authors:** Marcos Martínez‐Fernández, Yannic Hartmann, Bernd M. Schmidt

**Affiliations:** ^1^ Institut für Organische Chemie und Makromolekulare Chemie, Heinrich‐Heine‐Universität Düsseldorf Universitätsstraße 1 40225 Düsseldorf Germany

**Keywords:** Covalent organic frameworks, Metal‐organic frameworks, Porous materials, Porous organic cages, Self‐assembly

## Abstract

Confined nanospaces play a fundamental role in nature, inspiring synthetic analogues that emulate biological precision and efficiency. Among these, porous crystalline materials such as covalent organic frameworks (COFs), metal‐organic frameworks (MOFs), and molecular cage compounds have emerged as powerful platforms for catalysis, separation, and energy storage. Recent developments highlight the potential of porous organic cages (POCs) as modular building blocks for the construction of advanced materials. In this Minireview, their integration into extended frameworks, such as Cage‐COFs and Cage‐MOFs, is described, as they allow precise control over porosity and enhance chemical robustness. These hybrids merge the structural regularity of COFs with the discrete functionality of cages, enabling the design of lightweight, hierarchically organised materials. In addition, cage‐containing polymers and supramolecular frameworks are discussed. Collectively, these developments position POCs as versatile synthons for next‐generation porous materials, unlocking pathways toward functional, adaptive, and recyclable architectures.

## Graphical abstract

Confined nanospaces are key to the design of porous materials like COFs, MOFs, and molecular cages for applications in molecular recognition, catalysis, and energy storage. This Minireview explores the rising role of porous organic cages (POCs) as versatile synthons, highlighting their integration into Cage‐COFs, Cage‐MOFs, and Cage‐POPs. These hybrid materials offer tunable porosity, structural precision, and exciting potential for future applications.

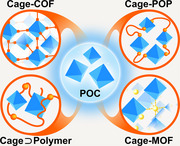

## 1 Introduction

The use of confined spaces at the nanoscopic level plays a central role in nature. Enzyme active sites and photosynthetic complexes that function within highly controlled local nanoscopic spaces use defined microenvironments to enhance reactivity and selectivity, efficient even under mild conditions.^[^ ^1^ ^]^ These natural systems have inspired the development of synthetic materials that replicate the spatial control found in biology.^[^ ^2^ ^]^ Among these, interconnected framework materials,^[^ ^3^, ^4^, ^5^, ^6^ ^]^ polymers^[^ ^7^ ^]^ and, recently, (supra)molecular cage compounds^[^ ^3^, ^8^, ^9^, ^10^, ^11^, ^12^, ^13^ ^]^ have emerged as highly versatile platforms.^[^ ^14^ ^]^ Their well‐defined pores or cavities, in combination with access by self‐assembly, make them ideal for applications in catalysis, gas storage, molecular separation, sensing, optoelectronics, and energy storage.^[^ ^15^ ^]^ By mimicking the structural precision and functional advantages of biological confinement, these materials offer new opportunities to control chemical processes at the molecular level. Metal‐organic frameworks (MOFs) and covalent organic frameworks (COFs) are the two most prominent classes of porous crystalline materials known for their high surface areas and tuneable structures.^[^ ^16^ ^]^

MOFs are constructed from metal ions or clusters coordinated to organic linkers, forming extended networks with precise pore architectures. Their modular design allows for fine control over chemical functionality.^[^ ^5^, ^6^ ^]^ In contrast, COFs are composed entirely of light elements such as carbon, hydrogen, nitrogen, and oxygen, linked through dynamic covalent bonds.^[^ ^3^ ^]^ This all‐organic composition gives COFs lower densities and elevated stabilities in some cases.^[^ ^17^, ^18^, ^19^ ^]^ Both frameworks exemplify the power of reticular chemistry in designing materials with tailored properties at the molecular level.^[^ ^16^ ^]^ In contrast to these extended networks, a rich chemistry for metal‐organic cages (MOCs) has been discovered in the last three decades.^[^ ^20^ ^]^

In the past two decades, porous organic cages (POCs), a relatively recent class of low‐density crystalline materials, started to provide a versatile platform for gas storage and separation, with potential applications in porous liquids,^[^ ^13^, ^21^ ^]^ high‐permeability membranes,^[^ ^22^, ^23^ ^]^ and heterogeneous catalysis.^[^ ^24^ ^]^ POCs are discrete molecules with intrinsic, guest‐accessible cavities, which may be augmented by extrinsic voids between the cages. To be porous in the solid‐state, their cavities must also be interconnected to yield a pore network. Self‐assembled POCs by dynamic covalent chemistry (DCC) or discrete molecular cages obtained by linear synthesis via irreversible covalent bond formation can serve themselves as building blocks for the assembly of supramolecular architectures. However, the main drawback is that the formed structure is not usually predictable, affecting the porosity of these materials, and the porous supramolecular framework normally displays high solubility or processability but low chemical and thermal stabilities, being prone to structural collapse. Recently, discrete organic cages and their derived supramolecular materials produced by hydrogen bonding, π–π interactions or van der Waals forces have garnered increasing interest. Despite their great applicability (vide supra), their structural integrity and function often rely on precise crystallisation conditions and weak interactions, limiting their robustness and scalability.^[^ ^16^, ^17^, ^18^, ^19^, ^20^, ^21^, ^22^, ^23^, ^24^ ^]^

The hybridisation of POCs and framework materials allows: i) the translation of host‐guest and solution studied chemistry to the solid‐state; ii) the partition of space or the introduction of new types of pores in extended solids and new diffusion pathways; iii) the pre‐designability of the cage pore (specially for amorphous materials); iv) the inhibition of vertex‐to‐cavity packing of molecular POC powders which lead to non‐porous structures; v) the reduction of interlayer interactions, increasing processability by exfoliation for 2D materials; and vi) the production of smaller pores often useful for gas separation. In addition, these systems, the porosity, mechanical stability, and chemical robustness are strongly influenced by both the intrinsic properties of the cage and the conditions of the assembly process, which also can be modulated by rational design, which is also laid out in two recent reviews.^[^ ^25^, ^26^ ^]^

## 2 Covalent Organic Frameworks

**Figure 1:**
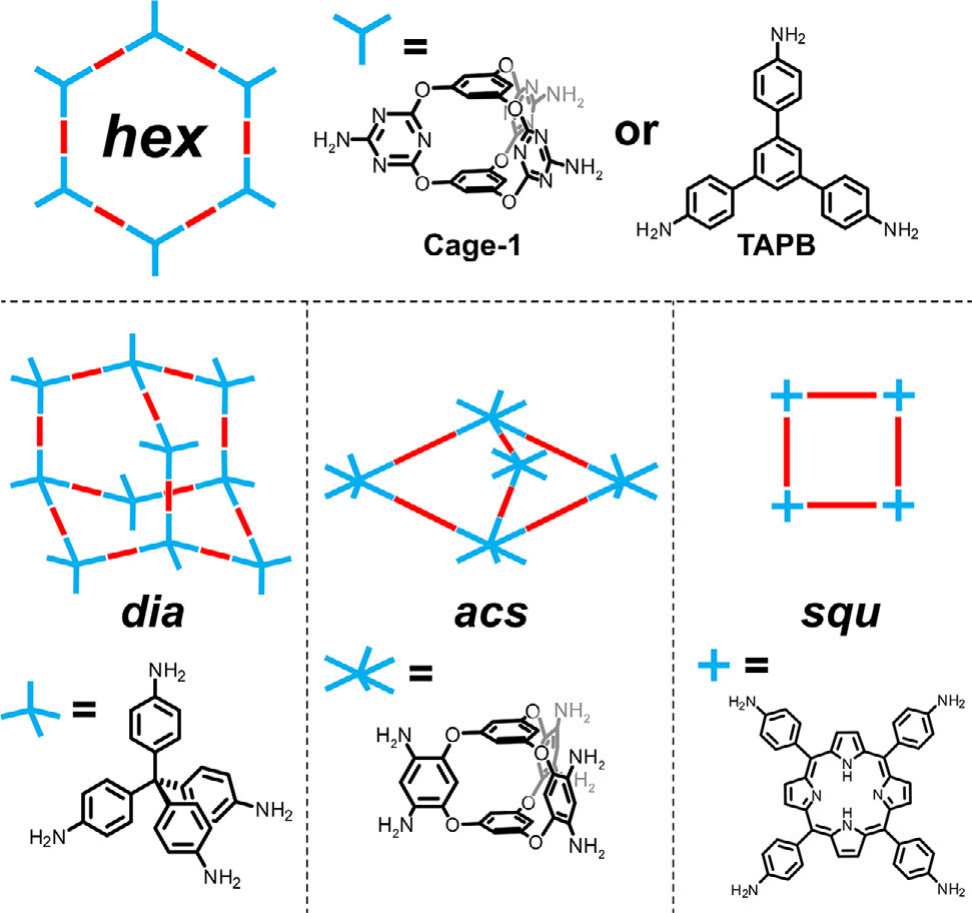
Schematic representation of topology diagrams showing different linkers (red bars represent terephthalaldehyde).

Out of all the frameworks discussed in this review, constructing COFs with narrow pore distributions using cages remains challenging due to the symmetry and structural requirements needed for employing POCs as monomers. To favour the obtainment of crystalline phases (or COF phases), it is necessary to employ highly symmetric linkers (Figure 1) with fixed orientations of the reacting functionalities and shapes to produce the extended, consistent and regular framework, which is not always the case in the POC field. Thus, the construction of cage‐based crystalline polymers promises access to novel materials by leveraging the well‐defined structures formed through the hierarchical arrangement of POCs. In this regard, COFs stand out as a promising candidate for the construction of cage‐based materials.^[^ ^17^ ^]^ First described by O. M. Yaghi and colleagues in 2005,^[^ ^27^ ^]^ these frameworks are typically synthesised through reversible bond formation using carefully designed linkers, resulting in fully organic 2D or 3D networks stabilised by covalent bonds and supramolecular interactions.^[^ ^17^, ^18^ ^]^ By topological analysis of the employed linkers combined with in silico calculations, the structural characteristics of the obtained frameworks, such as pore geometry, size, surface area, and, in some cases, even crystal lattices, can be predicted.^[^ ^28^ ^]^

Analogous to POCs, COFs are also obtained by the reaction of organic linkers, often employing reversible reactions, such as boronic ester, imine, or imide condensations. Thus, following the principles of DCC, the thermodynamic product is favoured, ideally yielding crystalline phases.^[^ ^29^ ^]^ Using these principles, a wide range of COF structures can be accessed, with topologies ranging from more common ones, e.g., *hex*, *sqr*, and *dia* (Figure 1), to more exotic ones like *the* or *bor*.^[^ ^28^ ^]^ However, utilising POCs as building blocks for COF formation introduces new paradigms in the design principles of these framework materials. Hypothetically, the construction of Cage‐COFs could be achieved by a sequential or a one‐pot synthesis.

2D‐COFs are characterised by their periodic bidimensional structure, which produces a porous 3D crystal by π–π interactions along the *c* axis.^[^ ^17^, ^18^, ^28^ ^]^ The neighbouring layers can present aligned disposition, also known as an eclipsed configuration (or AA stacking). The introduction of bulky moieties^[^ ^30^ ^]^ or additional interlayer interactions^[^ ^31^ ^]^ can lead to the displacement of these layers into more exotic staggered AB or ABC lattices with smaller pore sizes. In addition, intermediate situations with small offsets or rotations of one layer with reference to the others have also been found.^[^ ^29^ ^]^ On the other hand, most of the 3D‐COFs exhibit highly interpenetrated structures, which usually limits the pore size pre‐designability and makes the exact characterisation of these frameworks a great challenge.^[^ ^17^ ^]^

Cage‐based linkers lead to Cage‐COFs with minimal π–π interactions between the adjacent layers due to the unique 3D symmetries inherent to the cage linkers, in contrast to the more commonly used established COF monomers (Figure 1). This usually produces ABC lattices for 2D‐COFs^[^ ^32^ ^]^ and reduces the interpenetration possibilities up to three for the reported examples, facilitating the structural elucidation and giving rise to more pre‐designable pores.^[^ ^33^ ^]^ In addition, their defined cavities promote hierarchical complexity within the porous domain, allowing for the straightforward creation of different types of pores that contribute to building up the framework.

### 2.1 Sequential Synthesis of Cage‐COFs

**Figure 2:**
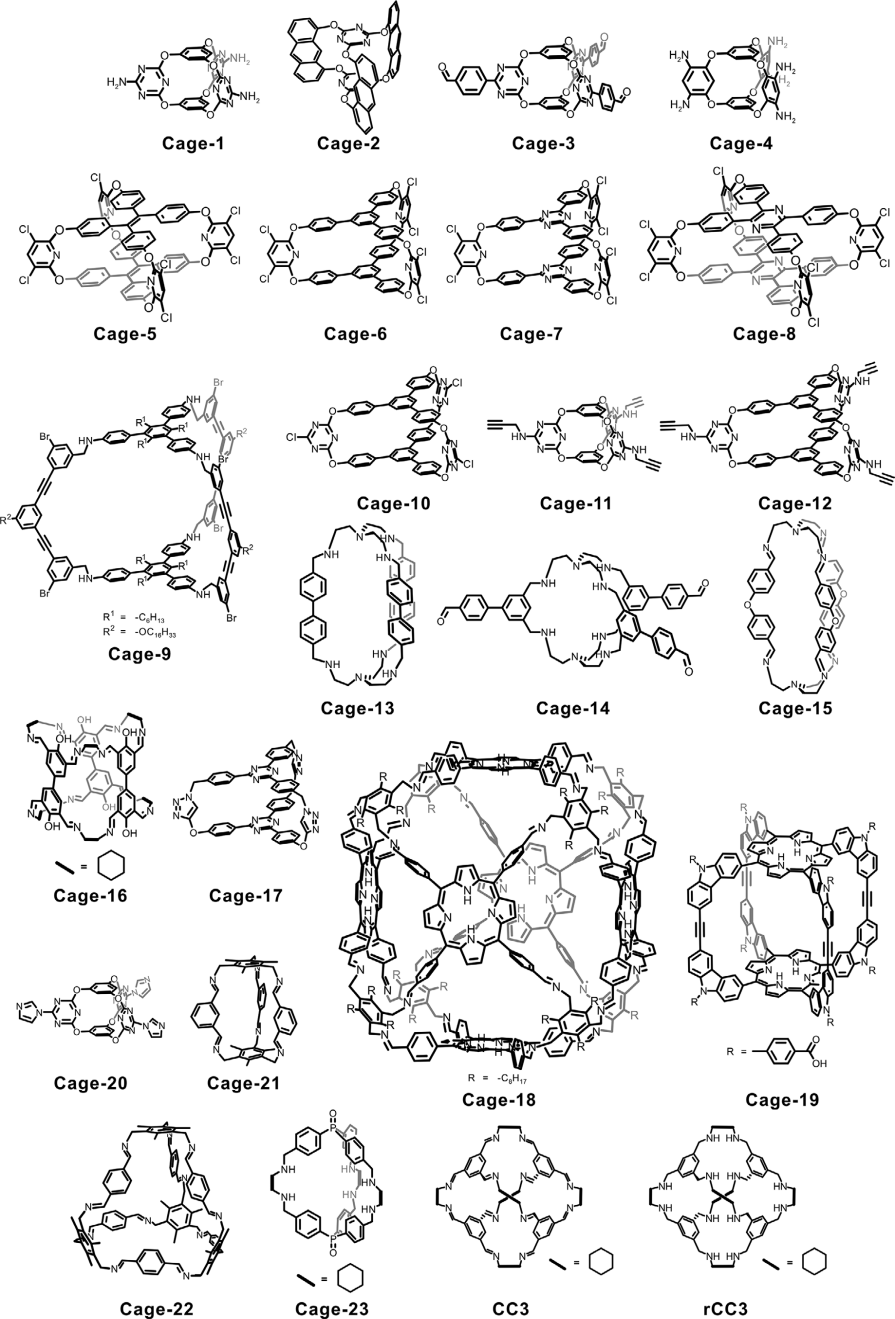
Overview of the cages discussed in this review.

2D‐Cage‐COFs are synthesised via the reaction of a preformed cage with different linkers, resulting in crystalline and porous frameworks. However, comparing conventional COF building blocks, such as tris(4‐aminophenyl)‐1,3,5‐benzene (TAPB) or 2,4,6‐trisamino‐triazine (TAT) and cages of similar symmetry, powder X‐ray diffraction (PXRD) and porosity analysis reveal that the resulting phases are entirely different. At first glance, Cage‐1 (*D_3h_*, Figures 1 and 2), TAPB (*D_3h_*), or TAT (*D_3h_*) copolymerised with a *C_2_* linker might appear to form similar hexagonal (*hex*) networks (Figure 1). On the one hand, TAPB or TAT in combination with terephthalaldehyde (TA) yield more likely eclipsed phases. On the other hand, when employing POCs with three‐dimensional structures, the obtained thermodynamic products are usually staggered ABC phases, probably due to the 3D configuration of these linkers. In addition, the experimental pore size distributions (PSD) of TAPB‐TA‐COFs are around 2.7 nm,^[^ ^29^ ^]^ while the ones found for Cage‐1‐TA‐COF (where Cage‐1 displays similar sizes to TAPB) have a calculated PSD of 1.1 nm due to the different phases produced.^[^ ^34^ ^]^

#### 2.1.1 2D‐Cage‐COFs

**Figure 3:**
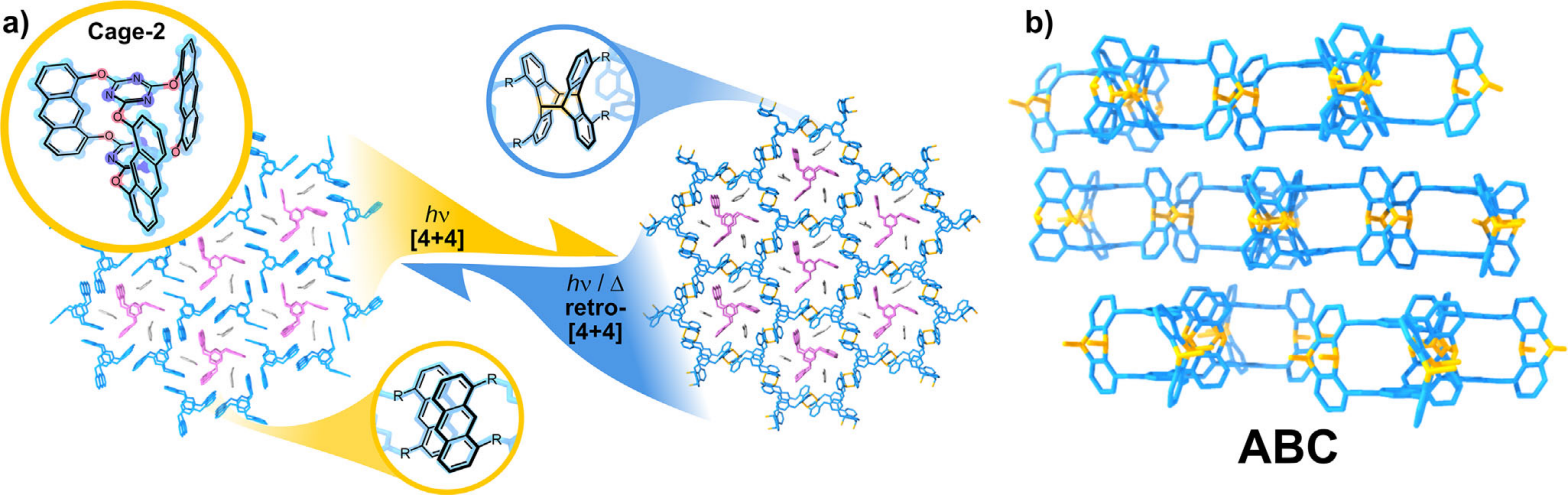
a) Left: top view of the crystal structure of Cage‐2 (the inlet depicts the structure of Cage‐2 and the antiparallel disposition of the anthracene moieties); right: top view of Cage‐COF‐2 showing the dimerised anthracene units); b) side view of Cage‐2‐COF showing ABC stacking configuration.^[^ ^35^ ^]^

The first and most distinctive Cage‐COF was reported by A. D. Schlüter's research team in 2014.^[^ ^35^ ^]^ The central cage (Cage‐2) used features anthracene panels, leading to cages with elongated internal cavities. The authors successfully obtained single‐crystals of the anthracene Cage‐2, which were subsequently transformed into the cage framework through photopolymerisation (Figure 3a). Following this strategy, the authors reported the first C─C‐linked single‐crystalline COF reported, to the best of our knowledge. The Cage‐2‐COF was obtained as polyhedra of >200 µm, and its solid‐state structure was unambiguously elucidated by single‐crystal X‐ray diffraction (SC‐XRD), confirming the anthracene [4 + 4] cycloaddition (Figure 3b). However, some precursor cages remain trapped within the pores, as evidenced by diffraction experiments. Thus, the theoretical porosity of this system is compromised by the design itself. In addition, the authors managed to isolate the 2D sheets by liquid‐phase exfoliation (LPE) assisted by *N*‐methylpyrrolidone, obtaining sheets of 10 µm of lateral extension but with heights around 2 nm, which corresponds well with few‐layered materials. Finally, the team also explored the thermal depolymerisation of the Cage‐COF at 180 °C, showing that the framework material could be transformed again into the discrete monomers, which could be repolymerised into the extended structure, showing the recyclability of these materials.

Another interesting example of 2D‐Cage‐COFs was reported by Q.‐Q. Wang et al. in 2019.^[^ ^32^ ^]^ The team crystallised the novel Cage‐COFs by reacting Cage‐3 with *p‐*phenylenediamine (PA) and 4,4‐biphenyldiamine (BPA) under solvothermal conditions, yielding Cage‐3‐PA‐COF and Cage‐3‐BPA‐COF, respectively. They not only demonstrated the potential to enhance hierarchical organisation within the COF paradigm by achieving double‐pore framework materials but also observed a unique ABC stacking phase. Adjacent layers seem to exhibit minimal π‐interactions, feature triangular pores, and maintain open interlayer spaces of approximately 1 nm, characteristics that closely resemble those of 3D‐COFs (vide infra), merging the borderline of 2D‐ and 3D‐COFs. In addition, the composition and orientation of the pore walls in these systems are worth highlighting. Unlike conventional COFs, where the C─H groups of the building blocks point toward the pore interior, in these Cage‐COFs they are aligned along the pore walls. This distinctive arrangement could contribute to enhanced porosity in the materials.^[^ ^36^ ^]^ The highest surface area was obtained for the Cage‐3‐PA‐COF (1237 m^2^ g^−1^), with a narrow pore size distribution of 1.1 nm. The molecular congener is non‐porous (4 m^2^ g^−1^), which highlights the successful strategy to obtain cage‐based, highly porous structures. In addition, the obtained framework materials were tested as CO_2_ adsorbents due to the great abundance of Lewis basic atoms (N and O), obtaining loadings of 43.8, 22.3 cm^3^ g^−1^ (for Cage‐3‐PA‐COF), and 37.3, 20.6 cm^3^ g^−1^ (for Cage‐3‐BPA‐COF) of CO_2_ at 273 and 298 K and 1 bar, respectively.

**Figure 4:**
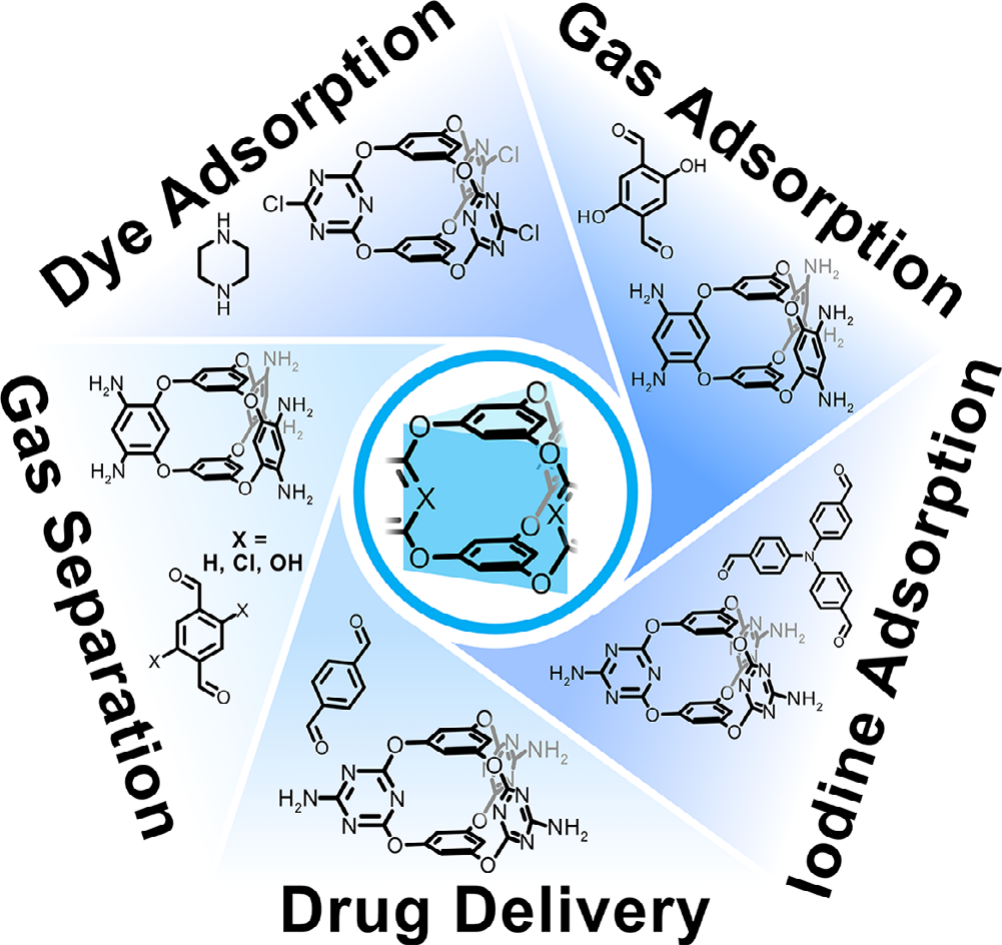
Overview of Cage‐COFs via sequential synthesis, highlighting potential applications.

Similarly, Z. Shi, S. Feng and colleagues reported Cage‐COFs by dynamic imine condensation between amine‐functionalised Cage‐1 and TA under solvothermal conditions, yielding Cage‐1‐TA‐COF (Figure 4).^[^ ^34^ ^]^ The obtained framework displayed similar structural features to Cage‐3‐PA‐COF and Cage‐3‐BPA‐COF, with an ABC‐stacked lattice but smaller pores due to the smaller size of Cage‐1 in comparison to Cage‐3. Cage‐1‐TA‐COF showed a surface area of 672 m^2^ g^−1^ with a narrow PSD centred at 1.0 nm. In this case, the researchers employed the cage‐based material as a model platform to study drug release, including ibuprofen, 5‐fluorouracil, and captopril. Owing to the unique structural characteristics of Cage‐1‐TA‐COF, the material was able to reach a high drug loading rate (up to 0.22 g of ibuprofen, 0.25 g of 5‐fluorouracil, and 0.30 g of captopril per gram of Cage‐COF). In addition, UV–vis spectrophotometry revealed well‐controlled release profiles, underscoring the broad versatility of Cage‐COFs for advanced biomedical applications.

P.‐Z. Li and Y. Zhao's research teams succeeded in crystallising a family of Cage‐COFs based on the reaction of Cage‐1 with different linear dialdehydes: TA, 4,4‐biphenyldicarboxaldehyde (BA), and 4,4″‐*p*‐terphenyldicarboxaldehyde (TDA), yielding Cage‐1‐TA‐COF, Cage‐1‐BA‐COF, and Cage‐1‐TDA‐COF, respectively.^[^ ^37^ ^]^ Consequently, they obtained isostructural frameworks with ABC stacked structures featuring larger pore sizes due to the use of larger aldehyde linkers. The surface areas were calculated to be 154, 174, and 181 m^2^ g^−1^ for Cage‐1‐TA‐COF, Cage‐1‐BA‐COF, and Cage‐1‐TDA‐COF, respectively, with narrow PSDs. In this study, the materials were tested as iodine vapour adsorbents by exposing the samples to the halogen gas at 77 °C. The iodine uptakes were determined by gravimetric techniques, obtaining maximum capacities of 262, 242, and 131 wt% for Cage‐1‐TA‐COF, Cage‐1‐BA‐COF, and Cage‐1‐TDA‐COF, respectively, which was rationalised in terms of the higher density of the adsorption sites (nitrogen in this case) for the COF with the smaller pores.

Finally, additional contributions should also be mentioned, such as the Cage‐COFs by X. Gui, K. Xu and collaborators,^[^ ^38^ ^]^ P. Li, Y. Zhao et al.,^[^ ^39^ ^]^ or M. Li and colleagues.^[^ ^40^ ^]^ Despite the great properties of these frameworks, the porosity or crystallinity of the materials could be improved, highlighting the challenges of producing well‐defined Cage‐COFs.

#### 2.1.2 3D‐Cage‐COFs

**Figure 5:**
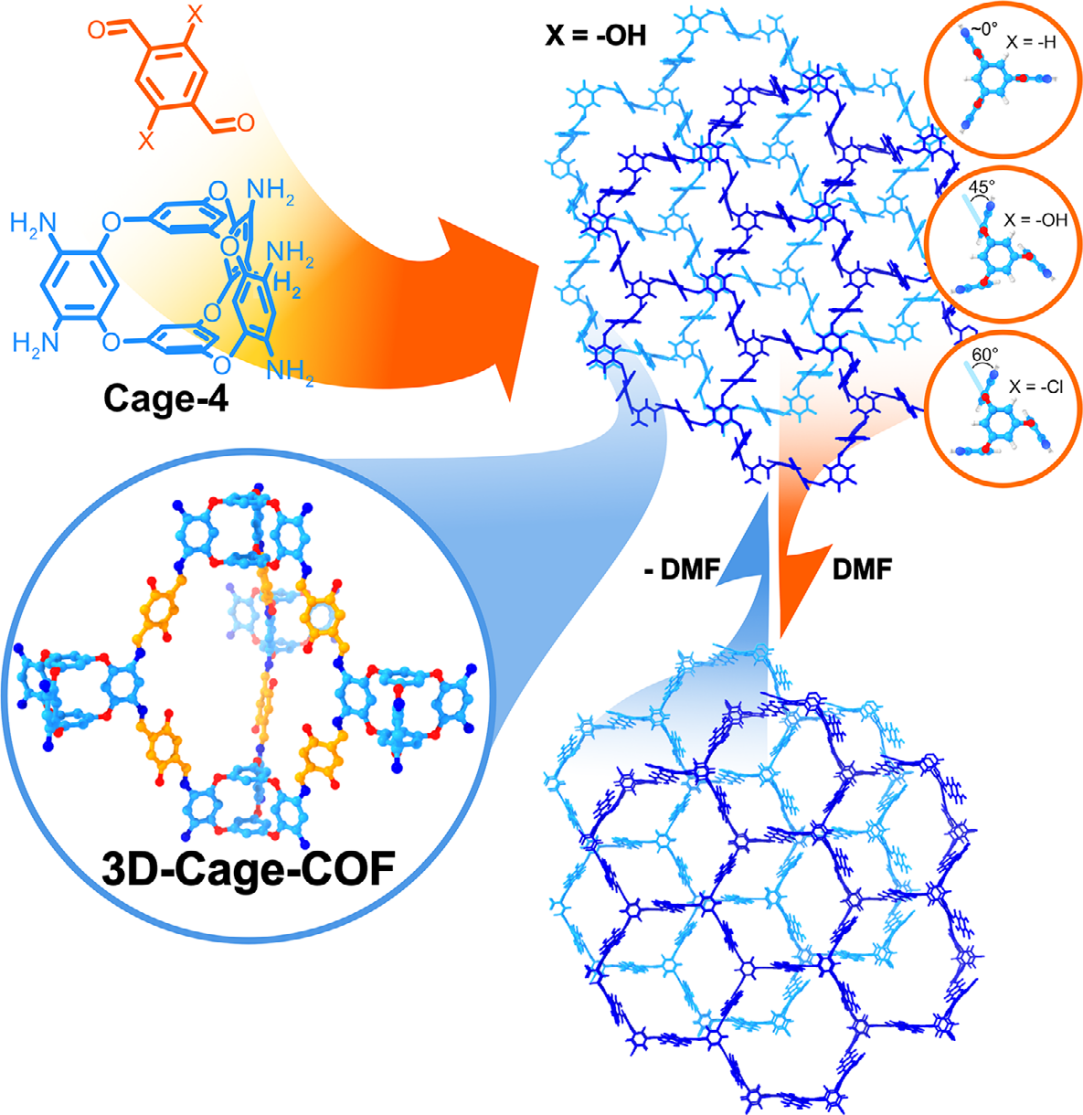
Synthesis of 3D‐Cage‐COFs by reaction of Cage‐4 with different dialdehydes and the dynamic adaptive geometry change behaviour upon exposure to DMF (the inlet depicts the torsion angles depending on the terephthalaldehyde substituents).^[^ ^33^, ^42^ ^]^

Inspired by the second 2D‐Cage‐COF, L. Chen, M. A. Little, and A. I. Cooper reported the first 3D‐Cage‐COF following the two‐step approach.^[^ ^33^ ^]^ They utilised a shape‐persistent organic cage, Cage‐4, which is structurally similar to Cage‐1 but exposes six pendant amine groups arranged in a trigonal prismatic configuration, rather than three amine groups in a triangular arrangement (Figure 4). After a solvothermal imine condensation reaction of Cage‐4 with 2,5‐dihydroxyterephthalaldehyde (DHTA), Cage‐4‐DHTA‐COF was obtained with an *acs* topology and 2‐fold interpenetrated structure. Compared to conventional linkers used in 3D‐COF synthesis, the incorporation of Cage‐4 reduces the likelihood of network interpenetration, limiting the maximum catenation to three, which facilitates the structural elucidation of these materials. In addition, the material exhibits reversible dynamic adaptive geometry change behaviour, characteristic of 3D‐COFs upon exposure to guests,^[^ ^41^ ^]^ such as DMF. Owing to the structural flexibility of the ether and imine functionalities in combination with the 3D structure, the framework undergoes a phase transition after the inclusion/removal of the guest molecules (Figure 5). With its high surface area (1037 m^2^ g^−1^), the material was tested as a CO_2_ and H_2_O adsorbent through the obtention of the respective gas sorption isotherms. Cage‐4‐DHTA‐COF captures 204 mg g^−1^ at 273 K and 1 bar and 107 mg g^−1^ at 298 K and 1 bar of CO_2_, surpassing chemically similar COFs, even some with greater surface areas, highlighting the beneficial structure of the 3D‐Cage‐COFs for adsorption applications. In addition, the sorption capacity of Cage‐4‐DHTA‐COF surpasses that reported for Cage‐3‐PA‐COF despite its lower surface area. This material stands out as a promising candidate for water harvesting since its adsorption capacity (22 wt% at p/p_0_) is comparable to the best‐performing COF (COF‐432) and shows excellent cycling performance.

Using the same design principles, two additional 3D‐Cage‐COFs were reported by the group of D. Yuan in 2021,^[^ ^42^ ^]^ by reacting Cage‐4 with three terephthalaldehyde derivatives bearing different heteroatomic substitutions at the positions 2 and 5 (─H, ─Cl, and ─OH). The solvothermal imine condensation reaction of Cage‐4 with TA yielded Cage‐4‐TA‐COF, the same 2‐fold interpenetrated framework with *acs* topology reported by A. I. Cooper et al.^[^ ^33^ ^]^ However, the reaction of the cage‐linker with DHTA and 2,5‐dichloro terephthalaldehyde (DCTA) yielded a contracted structure due to the strong non‐covalent interactions between the catenated frameworks (Cage‐4‐TA‐COF and Cage‐4‐DCTA‐COF, respectively). Calculations revealed an effect of the terephthalaldehyde substitution on the torsion angle of the diether cage arms, leading to slightly different unit cells of the frameworks. The degree of the average torsion angle (*D*_TA_), which was almost negligible for Cage‐4‐TA‐COF, was significantly increased to almost 45° for Cage‐4‐DHTA‐COF and 60° for Cage‐4‐DCTA‐COF (Figure 5). This effect, which was also corroborated by single‐crystal analysis of the respective molecular analogues, demonstrated the possibility of tuning the structural features of the framework via molecular design. The material's characterisation was completed with N_2_ sorption isotherm analysis, which revealed that the most rigid structures possess the highest surface areas, being 1143 m^2^ g^−1^ for Cage‐4‐TA‐COF, 923 m^2^ g^−1^ for Cage‐4‐DHTA‐COF, and 660 m^2^ g^−1^ for Cage‐4‐DCTA‐COF. Furthermore, the authors investigated the framework materials for the selective adsorption of CO_2_ over CH_4_. In addition, a similar example was reported by S. Wei, W. Lyu, X. Lu, and colleagues.^[^ ^43^ ^]^ In their work, a lithium‐decorated Cage‐COF showed enhanced CO_2_ adsorption (236 cm^3^ g^−1^) in comparison to the metal‐free counterpart (89 cm^3^ g^−1^) at 273 K and 1 bar.

### 2.2 One‐Pot Synthesis of COFs with Cage‐Like Cavities

**Figure 6:**
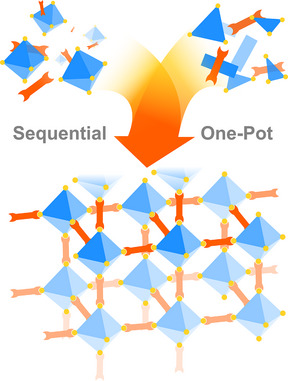
Representation Cage‐COFs formation versus COFs with cage‐like cavities. POCs are represented as blue octahedra, linkers in orange.

The main drawback of the sequential synthesis is the small sizes of the POCs employed for the Cage‐COF formation. This is presumably due to the symmetry requirements (see 2.1 and Figure 1) for COF crystallisation, especially for 2D‐COFs, where the extension of the covalent network is perpendicular to the interlaminar interactions (such as π–π contacts between the adjacent layers or other supramolecular forces).^[^ ^29^ ^]^ In addition, the synthesis of the Cage‐COFs has predominantly followed a strategy where pre‐synthesised cage building blocks react under thermodynamic conditions to yield the crystalline framework. It should be noted that all the POCs employed as linkers in the sequential synthesis are often covalent cages, which are just a small fraction of the expanding POC research field.^[^ ^12^, ^44^ ^]^ However, DCC‐based POCs are often not suitable for direct Cage‐COF formation, as they might undergo undesired cage‐to‐COF transformations during the process or decompose into oligomeric or polymeric species.^[^ ^45^, ^46^, ^47^, ^48^ ^]^ An elegant solution to this challenge lies in a reaction where the POC entities and the COF skeleton are formed simultaneously, referred to as *one‐pot synthesis* in this review (Figure 6).

When revisiting the topology diagrams,^[^ ^29^ ^]^ most of the POCs do not possess the desired symmetry for building well‐ordered and consistent frameworks. Thus, we propose the one‐pot approach, where just considering the linker's relative symmetries, cage‐like cavities can be directly formed during the COF crystallisation by a topology‐directed synthesis^[^ ^49^ ^]^ or by the linkages of the framework.^[^ ^50^ ^]^ All these frameworks are part of the bigger classification of COFs known as 3D‐COFs, but they exhibit multicompartment structures, featuring distinct pore environments. The main characteristics of this special kind of 3D‐COFs are i) the framework must show heteropore composition, differentiating the COFs with cage‐like cavities from conventional 3D‐COFs; ii) the cage cavities should present three‐dimensional structures with heights above the benzene size, being the smallest cavities in the framework; iii) the geometry of the obtained cage‐like cavities must resemble the reported cages. Thus, the incorporation of the desired cavities can be achieved, avoiding the sequential synthesis problems, including purification, high dilution conditions or the incorporation of *exo*‐functionalities. Despite these materials not being formally considered as Cage‐COFs since POCs are not directly used as linkers, such spatial segregation could still offer enhanced performance such as selective molecular adsorption, improved separation by differentiated diffusion pathways, site‐specific catalysis, and synergistic effects arising from the presence of varied cavity types.^[^ ^51^ ^]^ For the successful one‐pot synthesis of 3D‐COFs incorporating cage‐like cavities, the linkers must not only meet these symmetry requirements but also enable the simultaneous assembly of both the central cage‐like motifs and the extended framework. Despite the works compiled below not being self‐classified as Cage‐COFs, we envisage that they fit perfectly in the scope of this review, pushing the COF design rules boundaries one step further.

**Figure 7:**
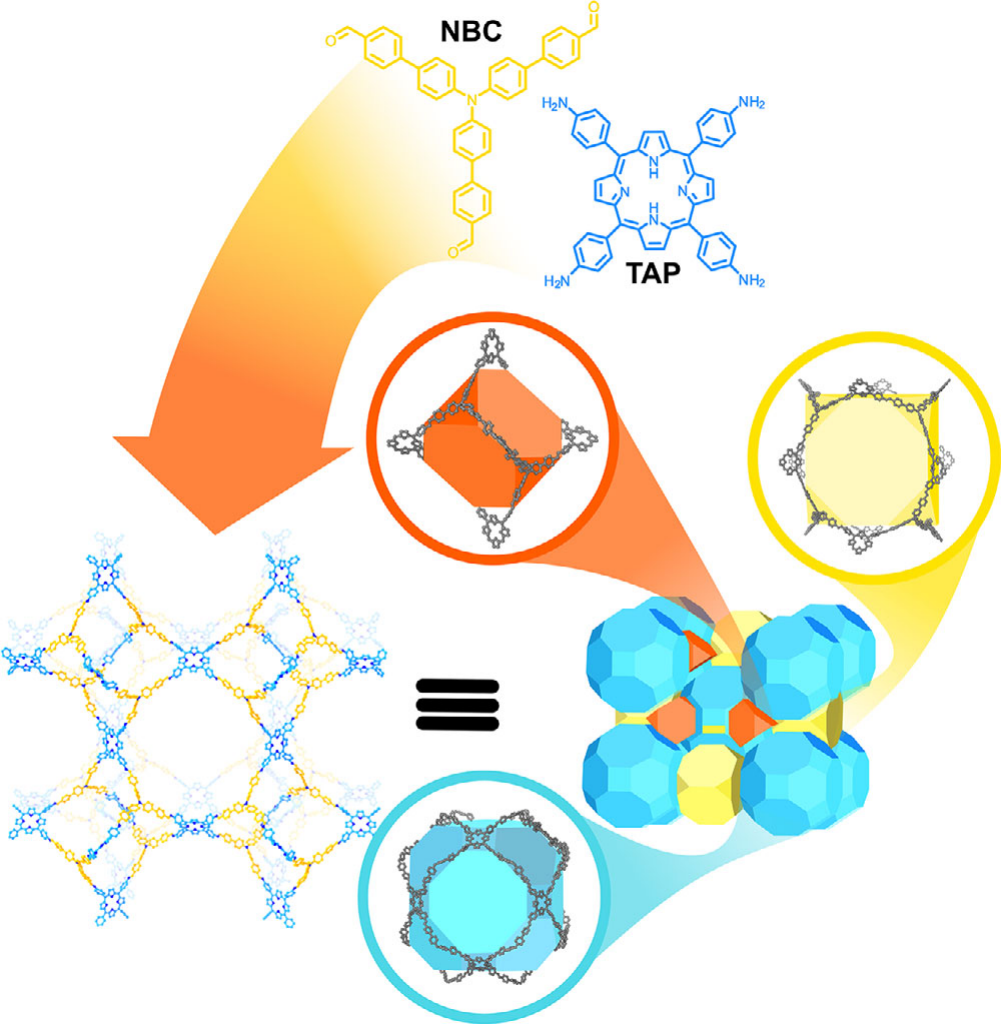
3D‐COF showcasing intrinsic cage‐like cavities due to the unique *tbo* topology, the chemical structures, and the lattice with the location of the different cavities.^[^ ^49^ ^]^

The first example of this kind of framework material was described by O. M. Yaghi and co‐workers in 2007, who obtained a *bor*‐based topology by the reaction of the tetrahedral tetra(4‐dihydroxyborylphenyl)methane (TBPM) and its silane analogue (TBPS) with triangular hexahydroxytriphenylene (HHTP).^[^ ^52^ ^]^ The reaction between TBPM and HHTP produced a *ctn* topology, while the reaction between TBPS and HHTP yielded a new type of COF topology characterised by tetrahedral structures joined together through the vertices (Si atoms) and organised in a cubic lattice. The cage‐like structure incorporates two different types of pores around 15 Å (for the cage‐like cavities) and 30 Å (mesopores of the COF), as demonstrated by a combination of PXRD and N_2_ sorption experiments. The cage‐like design enabled the formation of an ultralight structure with a density of 0.17 g cm^−3^, which is 15% less dense than the *ctn* isomer and contrasts with the densities calculated for MOFs with similar surface areas (0.56 g cm^−3^). Although this initial example was not further explored, it laid the foundation for the concept of compartmentalising space using cage‐like cavities. In 2008, O. M. Yaghi's group^[^ ^53^ ^]^ reported the assembly of tetraphenylmethane tetraboronic acids and *tert*‐butylsilane to yield triolborosilicate cage‐like cavities with a tetrahedral shape forming the extended framework. The so‐called COF‐202 presented a *cnt* topology (3D) elucidated by PXRD, supported by theoretical modelling. Furthermore, the material presented an extremely high surface area of 2690 m^2^ g^−1^. Despite the material not being further studied in applications, this work demonstrated the use of reticular chemistry to produce advanced structures with control of the topology of these systems at an atomic level. A further example was reported by Y. Liu and Y. Cui in 2018.^[^ ^49^ ^]^ They synthesised a *tbo* Cage‐COF by dynamic imine condensation of 5,10,15,20‐tetra(4‐aminophenyl)porphyrin (TAP) and 4′,4′″,4′″″‐nitrilotris[(1,1′‐biphenyl)‐4‐carbaldehyde] (NBC), yielding a cubic lattice of truncated tetrahedral structures of sizes around 14 Å, bridged together through the porphyrin monomers, featuring distinct pores intrinsic to this unique topology (Figure 7). Smaller truncated cubic pores, measuring 20 Å in size, are formed by four NBC linkers and twelve amine groups from four peripheral porphyrins. In contrast, larger cuboctahedral pores, with a diameter of 33 Å, arise from the assembly of twelve TAP monomers and forty‐eight aldehyde groups. Compared to the first *bor* topology reported by Yaghi,^[^ ^52^ ^]^ this structure is completely constructed from planar monomers and exhibits a smaller cage‐like cavity (around 15 Å). The periodical structure was studied by PXRD, revealing that the crystallisation results in a non‐interpenetrated *tbo* topology. In addition, the accessibility and exclusivity of different guests were studied by confocal microscopy, demonstrating the importance of the topological design. An identical framework was synthesised with a metallated porphyrin since the presence of the metal centres in the Cage‐COF structure can provide access to single‐atom catalysts (SACs). Both frameworks were investigated for the photocatalytic hydroxylation of arylboronic acids, as well as the defluoroalkylation of trifluoromethyl aromatics with alkenes, showing good yields, functional group tolerance and high reusability.

Twelve years later, in 2020, O. M. Yaghi's research team further continued the one‐pot strategy for obtaining COFs with cage‐like cavities via condensation of phenylphosphonate boronic acid derivatives to yield cubic borophosphonate COFs.^[^ ^50^ ^]^ The solid‐state structure was elucidated by PXRD data, confirming the extended cubic structure based on the *bcu* topology. Additionally, the authors showcased the adaptability of the condensation process to create borophosphonate structures using various commercially available linkers, resulting in frameworks with varying pore sizes. In this case the material was studied as a potential H_2_ adsorbent, revealing a gravimetric uptake of 11.6 mg g^−1^ at 1 bar and 77 K. More recently, Y. Zhao, T. Ben, and Y. Negishi's research teams explored innovative topologies for accessing new 3D‐COFs with cage‐like cavities based on already reported structures in the Reticular Chemistry Structure Resource database.^[^ ^54^ ^]^ Using dynamic imine condensation between tris‐(4‐aminophenyl)‐*s*‐triazine and the 4′,5‐bis(3,5‐diformylphenyl)–3′,6′‐dimethyl‐[1,1:′2′,1′‐terphenyl]−3,3′′,5,5′′‐tetracarbaldehyde, the authors were able to crystallise a previously unreported COF.^[^ ^55^ ^]^ This framework can be considered as imine‐based tetragonal structures linked together through *p*‐xylene bridges in a cubic lattice. The structural elucidation was carried out by PXRD, supported by theoretical calculations, revealing that the obtained materials present the *the* topology with 2‐fold interpenetration of the framework. In addition, the surface area was calculated to be 684 m^2^ g^−1^, showing CO_2_ adsorption to a maximum loading of 34.2 cm^3^ g^−1^ at 273 K and 1 bar, as well as iodine adsorption, where it demonstrated an outstanding sorption capacity of 6.6 g g^−1^.

Finally, despite being a macrocycle‐ and not a POC‐based superstructure, this last example deserves to be highlighted as an inspirational method for the development of novel COFs with cage‐like cavities using topological bonds.^[^ ^56^ ^]^ CdPoly[2]C is produced by poly‐catenation, which was reported recently by M. A. Olson, F. Gándara, and A. Trabolsi.^[^ ^57^ ^]^ To favour the poly‐catenation reaction, the authors leveraged the pre‐organisation of the monomers by forming a mixed coordination compound between the DAB‐NH and Tris‐DFP while carrying out the imine‐induced polymerisation. The resulting central motifs of the COFs are formed by the catenation of different DAB‐NH moieties, while Tris‐DFP represents the knots of the organic polymer. The formation of the periodic network was studied by PXRD in combination with in silico models, suggesting that the CdPoly[2]C adopts a honeycomb *hcb* topology with an ABC lattice as its most probable structure. In addition, they obtained a COF decorated with Cd(II) ions, which were removed to obtain a fully organic network, and in a consecutive step, the imine linkages were reduced to more robust amine groups. PXRD analysis of Poly[2]C showed broader diffraction reflexes, probably due to the loss of structural rigidity. The materials were studied as CO_2_ adsorbents, however, the uptake was low in comparison with other Cage‐COFs with bigger interlayer spaces.

## 3 Porous Organic Polymers

The formation of a porous structure is just one of many possible solid‐state arrangements for each cage. Dense packing of POCs often limits the utilisation of their intrinsic porosity, with the accessible surface area sometimes being dominated by extrinsic porosity arising from void spaces between the crystallised cages.^[^ ^58^ ^]^ A problem more often encountered is the collapse of the intrinsic pores when the incorporated solvent molecules are removed in vacuo with or without heat treatment.^[^ ^59^ ^]^ Therefore, the incorporation of POCs into extended polymers has been reported as a novel approach to inhibit dense packing or detrimental window‐to‐vertex packing.^[^ ^60^ ^]^ Porous organic polymers (POPs) can be differentiated from conventional polymers by their persistent and interconnected void spaces, which can “turn on” the porosity of POCs.^[^ ^8^ ^]^ This structural feature allows the permeation of guest molecules through a solid matrix, which is important for solid‐state applications.^[^ ^61^ ^]^

POPs are often obtained as insoluble materials, which bypasses the leaching of the POCs in heterogeneous systems. The synthesis of many POPs relies on irreversible covalent reactions, typically yielding amorphous materials with enhanced chemical and thermal stability due to the robustness of the covalent linkages, especially when compared to dynamic, reversible frameworks. In addition, POPs typically feature lightweight atomic compositions which, combined with their high surface areas, result in low‐density materials.^[^ ^61^ ^]^

Despite their outstanding properties and broad applicability, the unknown exact composition of POPs^[^ ^62^, ^63^ ^]^ hinders the establishment of structure‐property relationships. When assembling Cage‐POPs, the type of polymerisation reaction must be carefully considered to ensure compatibility with the cage, as solid‐state characterisation of POP‐based materials can be challenging. Moreover, the typically uncontrolled polymerisation often leads to broad pore size distributions, limiting pre‐designability at the nanoscale. However, incorporating well‐defined POCs into the POP matrix preserves the pre‐designed cage cavities, enabling selective guest adsorption and inter‐pore diffusion pathways not easily achieved with conventional monomers for separation or filtration applications.^[^ ^64^, ^65^ ^]^ Some authors already reported the interfacial polymerisation giving rise to Cage‐POP free‐standing films^[^ ^64^, ^66^ ^]^ or over substrates to obtain modified surfaces,^[^ ^67^ ^]^ pushing the frontiers of this emerging research field. To the best of our knowledge, there are two main strategies to produce Cage‐POPs. The first involves homocoupling reactions between cage monomers,^[^ ^62^, ^63^, ^68^ ^]^ the second relies on more‐component strategies, including cross‐coupling reactions or condensation reactions.^[^ ^59^, ^69^ ^]^ Under appropriate conditions, both methodologies can lead to the formation of amorphous porous solids.

### 3.1 Cage‐POPs via Homocoupling Reactions

An intriguing example reported by J. Liu, Y. Zheng, B. Tan, C. Zhang and colleagues is based on tetraphenylethene (TPE) cages (Cage‐5). This moiety is a well‐known unit to obtain solid‐state fluorescent materials by aggregation‐induced emission (AIE).^[^ ^59^ ^]^ The authors envisioned that CO_2_ could be trapped inside the TPE rotatable phenyl rings, resulting in an enhanced emission intensity. A nickel (0)‐catalysed Yamamoto‐type poly‐Ullman coupling reaction was used to directly link the Cage‐5, and the obtained Cage‐POPs presented a PSD from 1 to 10 nm and a high surface area (929 m^2^ g^−1^), which contrasts with the barely porous TPE cages (8 m^2^ g^−1^). The CO_2_ uptake increased from 13.5 cm^3^ g^−1^ at (273 K and 1.0 bar) and 6.9 cm^3^ g^−1^ (at 298 K and 1.0 bar) to 49.3 cm^3^ g^−1^ (at 273 K and 1.0 bar) and 28.4 cm^3^ g^−1^ (at 298 K and 1.0 bar). In addition, this material was employed as CO_2_ sensor due to the blockage of the ring rotation produced by CO_2_ in the AIE cage.

The team also reported a similar system based on trigonal cages 6 and 7.^[^ ^70^ ^]^ Two different Cage‐POPs were synthesised by a poly‐Ullman reaction to yield frameworks with similar structural features, with a PSD from 2 to 10 nm and increased surface areas from 18 m^2^ g^−1^ (pristine cages) to around 800 m^2^ g^−1^ for both Cage‐POPs. The formation of the extended networks also enhanced the CO_2_ adsorption capacities for both Cage‐POPs. Thus, Cage‐6‐POP and Cage‐7‐POP were 53.54 and 60.21 cm^3^ g^−1^ (at 273 K and 1.0 bar) and 30.05 and 34.41 cm^3^ g^−1^ (at 273 K and 1.0 bar), respectively. It is worth noting that the *s*‐triazine‐functionalised POP exhibited a higher adsorption capacity compared to the triphenyl‐1,3,5‐benzene derivative, attributed to the increased number of Lewis basic adsorption sites.

**Figure 8:**
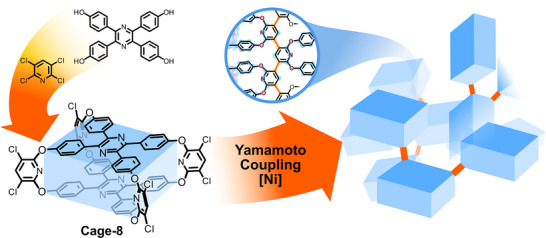
Synthesis of Cage‐8 and Yamamoto coupling to give Cage‐8‐POP. Cage‐8 is represented as blue prisms, the formed C─C bonds are represented by orange lines.^[^ ^69^ ^]^

Z. Wang, J. Bu, C. Zhang and colleagues synthesised a new Cage‐8‐POP based on the pseudo‐cubic Cage‐8 by a Yamamoto‐type Ullmann homocoupling reaction (Figure 8).^[^ ^69^ ^]^ Whereas the pure Cage‐8 was not porous (7 m^2^ g^−1^), the obtained polymers presented a high surface area of 929 m^2^ g^−1^ with micropores showing a PSD around 0.5 nm, which aligns well with the inner cavity of Cage‐8. This work substantiates that the cage‐to‐framework strategy is a viable approach to avoid the unfavourable dense packing of cages and “turning on” the porosity of the cage‐based materials. In addition, the obtained Cage‐POP showed great CO_2_ uptakes of 35.92 cm^3^ g^−1^ (at 273 K and 1.0 bar) and 25.27 cm^3^ g^−1^ (at 298 K and 1 bar) in contrast to pristine Cage‐8 (8.49 cm^3^ g^−1^ at 273 K and 1 bar) and 4.29 cm^3^ g^−1^ (at 298 K and 1 bar).

### 3.2 Cage‐POPs via Heterocoupling Reactions

W. Zhang and co‐workers reported the synthesis of the Cage‐9‐DEB‐POP via Sonogashira coupling between Cage‐9 and 1,4‐diethynylbenzene (DEB).^[^ ^63^ ^]^ The reported Cage‐9, featuring a trigonal prism shape, was obtained from dynamic covalent imine chemistry, followed by reduction of the imine bonds, ensuring the orthogonality to the polymerisation reaction. The cage‐to‐framework conversion was estimated to be around 50% by ^13^C solid‐state NMR. PXRD analysis revealed that the resulting framework was predominantly amorphous, with small crystalline regions due to the uncontrolled C─C cross‐coupling reaction. Notably, the obtained Cage‐9‐DEB‐POP showed increased thermal stability and increased CO_2_ uptake compared to its cage monomer. A series of cages was synthesised to further investigate CO_2_ uptake and selectivity over N_2_, demonstrating that the higher density of amines plays a key role in obtaining these properties. In a follow‐up study, the group reported the microwave‐assisted synthesis of novel cage‐POPs by Cage‐9 reaction with different diacetylenes with increased lengths or alkyl‐ether chains to study the effect of the co‐monomer in the polymerisation.^[^ ^62^ ^]^ The cage‐to‐framework conversion was estimated to be around 40% for all the Cage‐POPs through ^13^C solid‐state NMR. The authors demonstrated that Cage‐POPs exhibit increased CO_2_ and N_2_ uptake compared to the parent Cage‐9, with further enhancement observed when incorporating longer diacetylene derivatives. Conversely, while the N_2_ uptake of the Cage‐POP containing alkoxy chains was lower than that of the pristine cage, its CO_2_ uptake was improved due to favourable electrostatic interactions with the oxygen atoms embedded.

A. Coskun and colleagues utilised an S_n_Ar reaction of Cage‐10 with hydrazine (HZ), TAPB and tetra‐(4‐aminophenyl)‐adamantane (TAPA) to access Cage‐10‐HZ‐POP, Cage‐10‐TAPB‐POP and Cage‐10‐TAPA‐POP with different dimensions.^[^ ^68^ ^]^ The porosity of the obtained materials was studied by Ar sorption isotherms at 87 K, revealing surface areas of 629, 711, and 844 m^2^ g^−1^, respectively. The employment of three‐dimensional and larger linkers favours Cage‐POPs with a higher degree of porosity. These values surpass the surface area found for the cage‐linker (3 m^2^ g^−1^). The authors also investigated the CO_2_ uptake up to 1 bar at 273 and 298 K, revealing an increase in the CO_2_ uptake with the increase in the dimensionality of the co‐monomer. On the one hand, the CO_2_ adsorption was 3.55, 3.62, and 4.21 mmol g^−1^ at 1 bar and 273 K for Cage‐10‐HZ‐POP, Cage‐10‐TAPB‐POP, and Cage‐10‐TAPA‐POP, respectively. On the other hand, the polymers adsorbed 1.75, 1.96, and 2.26 mmol g^−1^ at 1 bar and 293 K, respectively. In addition, the CO_2_/N_2_ selectivity was measured to be 100.1, 84.7, and 72.2. This was also in good agreement with the Q_st_ 42.9, 37.3, and 34.4 kJ mol^−1^ for Cage‐10‐HZ‐POP, Cage‐10‐TAPB‐POP, and Cage‐10‐TAPA‐POP, which was rationalised in terms of the decreased cage densities within the framework and due to the presence of the acidic N─H bonds in the Cage‐10‐HZ‐POP. From this study, it can be concluded that the trade‐off between porosity, gas uptake, and adsorption site densities is an important factor to consider when designing novel materials.

O. Buyukcakir's research team reported the synthesis of Cage‐11‐TAPT‐POP and Cage‐12‐TAPT‐POP by Copper(I)‐catalysed Azide‐Alkyne Cycloaddition (CuAAC) polycondensation with 2,4,6‐tris(4‐azidophenyl)‐1,3,5‐triazine (TAPT).^[^ ^60^ ^]^ The calculated BET surface areas for Cage‐11‐TAPT‐POP and Cage‐12‐TAPT‐POP were 193 m^2^ g^−1^ and 423 m^2^ g^−1^, respectively. Both materials were tested as iodine vapour adsorbents, with maximum uptakes of 3.14 g g^−1^ for Cage‐11‐TAPT‐POP and 4.02 g g^−1^ for Cage‐12‐TAPT‐POP. Their adsorption capacity was also evaluated in aqueous KI/I_2_ solutions, where Cage‐11‐TAPT‐POP showed an uptake of 3.35 g g^−1^, while Cage‐12‐TAPT‐POP reached 2.24 g g^−1^. Interestingly, the maximum adsorption capacities in the gas and solution phases were inverted.

### 3.3 Cage‐POPs by Condensation Reactions

N. M. Khashab, S. P. Nunes, and co‐workers successfully synthesised Cage‐POP films with nanometre‐scale thickness through the interfacial polymerisation of Cage‐13 and reduced CC3 cage (rCC3).^[^ ^66^ ^]^ CC3 features an octahedral architecture, with four benzene rings at the vertices and twelve imine groups forming the edges of the polyhedron, and is arguably the most widely used POC in porous materials. It can be readily reduced to yield rCC3 using soft reducing agents.^[^ ^71^, ^72^, ^73^ ^]^ Integration into films was achieved via amide bond formation with benzene‐1,3,5‐tricarbonyl chloride (BTC) on a porous polyacrylonitrile (PAN) support, producing Cage‐13‐BTC‐POP and rCC3‐BTC‐POP. The obtained materials were amorphous since not all the amine groups reacted during the polymerisation, with the free amine functionalities estimated to be around 80% by TGA. The membranes were studied toward the permeability of different water‐soluble dyes and demonstrated that the permeance of the molecules is a function of the size of the cages employed in the polymerisation and the molecular weight of the molecule studied, even rejecting the diffusion of some dyes with the greatest sizes. Finally, Pd nanoparticles (NPs) encapsulated in rCC3‐BTC‐POP membranes showed excellent performance and recyclability for dye reduction. In 2024, Schmidt and co‐workers also reported membrane formation of fluorinated macrocycles and cages with 1,4‐diisocyanatobenzene at the interface of two immiscible solvents using their isocyanate‐induced azadefluorination cyclisation for cross‐linking.^[^ ^74^ ^]^

**Figure 9:**
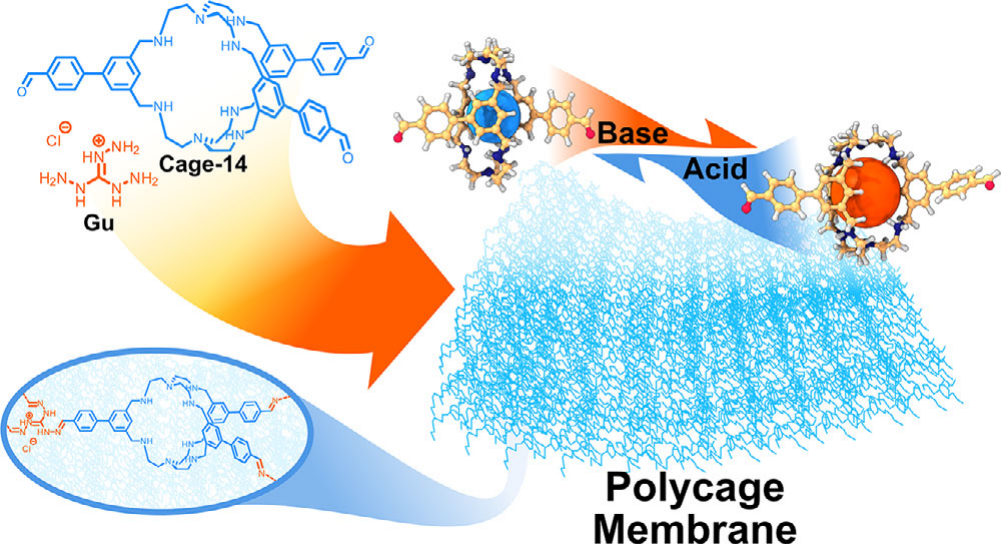
Synthesis of Cage‐14‐Gu‐POP highlighting the pH‐responsive cage (left contracted structure and right expanded one). The inlet represents the chemical structure of the Cage‐POP.^[^ ^64^ ^]^

Z. Lai and N. M. Khashab's research team then reported the synthesis of Cage‐14‐Gu‐POP while controlling the macroscopic form of the resulting polymer.^[^ ^64^ ^]^ They employed a tris(2‐aminoethyl)amine‐based Cage‐14 *exo*‐functionalised with aldehyde groups, which exhibited two distinct configurations depending on the pH of the medium. Through simple acidification or neutralisation, the cage expands or contracts, respectively, resulting in stimuli responsiveness (Figure 9). Polymerisation was carried out using dynamic imine polycondensation with triaminoguanidinium chloride (Gu) on a silicon surface, giving flexible free‐standing membranes. In addition, the group also varied the membrane's thickness by changing the concentration of monomers during the polymerisation, demonstrating the possibility of tailoring its dimensions from 12 to 48 µm. The surface area of the Cage‐POPs ranged from 69 to 16 m^2^ g^−1^ depending on the pH, with the PSD being centred at 0.4 nm for the neutralised polymer. Upon acidification, the material exhibited accessible pore sizes ranging from 0.6 to 1 nm, confirming its stimuli‐responsive behaviour. Given this pH‐dependent response, Cage‐14‐Gu‐POP was employed as an osmotic energy harvester. The results indicated that the acidic form of Cage‐14‐Gu‐POP achieved a maximum power density of 5.8 W m^−2^, which is four times higher than that of the neutralised membrane. Furthermore, these findings were validated using natural river and seawater, yielding comparable results.

X. Cao and colleagues also investigated rCC3 for the synthesis of rCC3‐BTC‐PP‐POP membranes using interfacial polymerisation of BTC and piperazine (PP) in different ratios to obtain an amide‐crosslinked polymer.^[^ ^67^ ^]^ The authors envisioned that Cage‐POP films with embedded nanopores could function as molecular sieves, permitting the passage of water molecules while rejecting organic pollutants. By adjusting the rCC3/PP ratio, they successfully fabricated films with thicknesses of approximately 160 nm, and amide bond formation was confirmed by FTIR and XPS. Physical characterisation using electron microscopy revealed a homogeneous membrane surface, attributed to intermolecular hydrogen bonding via N─H functionalities. Finally, water permeability and dye rejection tests (methylene blue and acid fuchsine) demonstrated that the synthesised rCC3‐BTC‐PP‐POP allows water permeation while effectively blocking organic dyes.

## 4 Cage⊃Polymers

**Figure 10:**
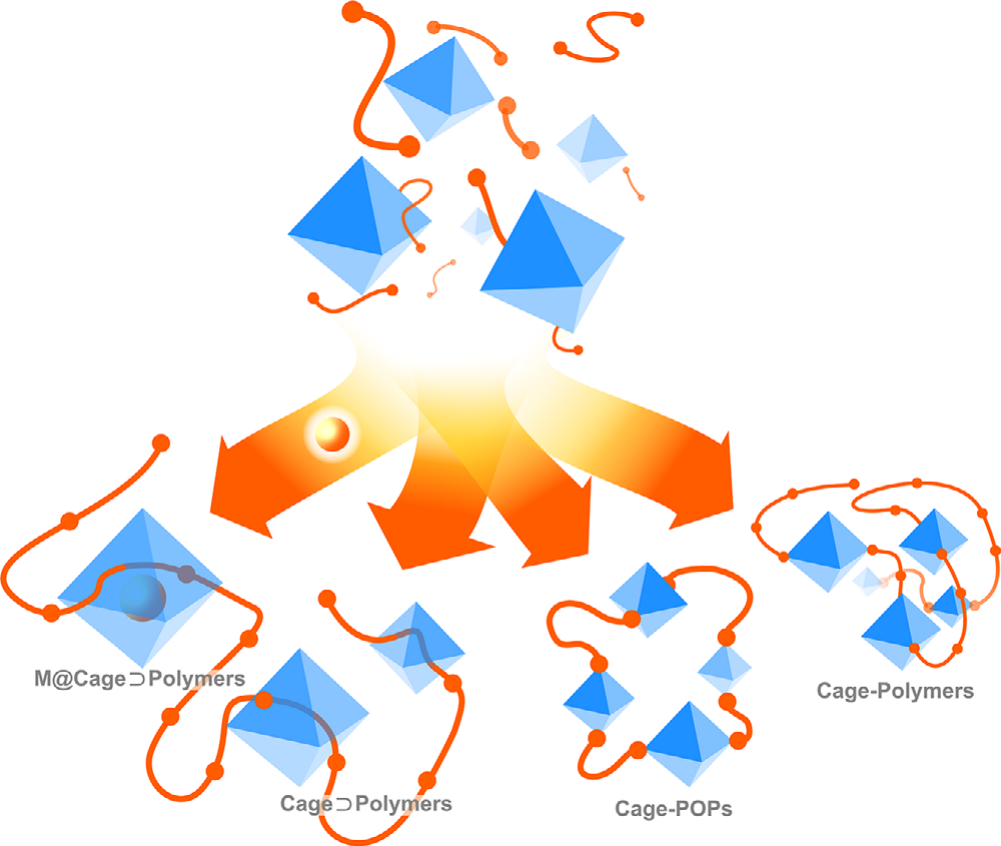
Approaches toward Cage⊃Polymer materials. POCs are represented as blue octahedra, and polymers are represented as orange chains.

Probably the most straightforward approach for obtaining POC superstructures is the physical mixture with well‐researched polymers, such as polymethyl methacrylate or polystyrene, to produce Cage⊃Polymer composites. The hybridisation of polymers with nanomaterials,^[^ ^75^, ^76^ ^]^ luminophores,^[^ ^77^, ^78^ ^]^ and antibacterial moieties,^[^ ^79^ ^]^ is widely employed in polymer science to modify macromolecular structures. POCs are ideal candidates for incorporation into polymeric structures since they allow for the introduction of well‐defined cavities into the polymer network,^[^ ^80^ ^]^ which can be exploited to accommodate specific guest molecules.^[^ ^81^ ^]^ In addition, external cavities can be generated during the assembly of these materials due to the inefficient aggregation of the POC/polymer interphase,^[^ ^22^ ^]^ providing diffusion channels. Polymers furthermore inhibit the dense packing of POCs into non‐porous structures,^[^ ^80^ ^]^ maximising accessible porosity. In addition, the processable nature of thermoplastics or soluble polymers could allow moulding of the Cage⊃Polymer composites into tailor‐made materials such as flexible films,^[^ ^81^ ^]^ thin, or mixed matrix membranes^[^ ^82^ ^]^ or solid‐state polymeric electrolytes.^[^ ^83^ ^]^ Some methods for producing these materials could be: i) polymerisation in the presence of the POCs,^[^ ^80^ ^]^ which could produce mechanical interconnection between POC and polymer ii) solid mixture,^[^ ^83^ ^]^ and iii) solution/drop‐casting, which guarantees the homogeneous mixture of POCs with the host polymers (Figure 10).^[^ ^22^ ^]^

### 4.1 Synthesis of Cage⊃Polymers

**Figure 11:**
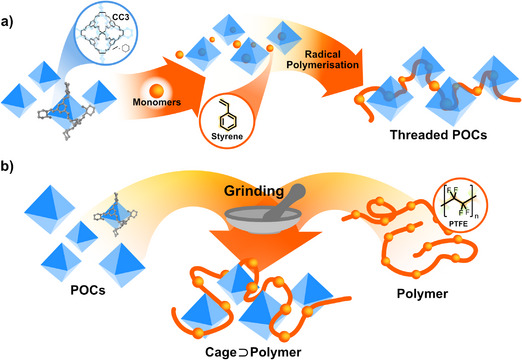
Approaches toward Cage⊃Polymers with POCs represented as blue octahedra, and polymers are represented as orange chains. a) Adsorption of a monomer which is subsequently polymerised, b) schematic representation of cage⊃polymer production by physical mixture highlighting the grinding process.

T. Uemura and co‐workers reported the first radical polymerisation within a POC, using vinyl monomers in the presence of Cooper's CC3 cage to afford CC3⊃PolySt. This host‐in‐host material was thus obtained employing a bottle‐around‐ship strategy.^[^ ^80^ ^]^ Building on the intrinsic microporosity and guest uptake enhancement of the POC in its amorphous state, volatile guest molecules of the CC3 crystals were removed to obtain an amorphous material, which was subsequently loaded with polymerisable styrene monomers (St), followed by radical polymerisation, yielding the amorphous CC3⊃PolySt (Figure 11a). It should be noted that along with the protocol, involving first adsorption of styrene and a second stage of polymerisation, produces the threading of the POC structures, preventing leaching. In addition, the Khashab group also explored the formation of composites by co‐dissolving Cage‐15 and polyvinylidene difluoride (PVDF) and subsequent evaporation,^[^ ^81^ ^]^ where the exposure of CC3⊃PVDF to organic solvents induced reversible stretching and bending behaviour due to the interaction of the POC with the vapour molecules.

An approach toward Cage⊃polymer catalysts was undertaken by J. Sun and J. Yuan in 2022,^[^ ^84^ ^]^ aiming for the implementation of Au NPs for enzyme catalysis. Despite the excellent catalytic properties of metal NPs,^[^ ^85^, ^86^ ^]^ their rapid agglomeration due to thermodynamic instability often limits their applicability by reducing recyclability. To address this issue, metal NPs are often immobilised in solid matrices.^[^ ^87^, ^88^ ^]^ The cationic rCC3•HCl, derived from rCC3, followed by protonation of the amine groups, was used as a host for the nanoconfinement of Au NPs. Au NPs were synthesised in situ within the ionic cages, yielding Au@rCC3•HCl. Using cages as hosts offers a promising strategy, as confinement controls NP growth, leading to uniform distribution, which is crucial for establishing structure‐property relationships in applications such as electrocatalysis.^[^ ^89^ ^]^ Replacing the Cl^−^ counter anions with 4‐styrenesulfonate (SS) anions produced the linker Au@rCC3•HSS, which was then used to synthesise Cage⊃Polymers by styrene polymerisation. For comparison, the metal‐free rCC3•HCl was similarly converted into the polymerisable rCC3•HSS using the same protocol. Each cage has an octahedral shape and is surrounded by 12 polymerisable counter anions. Both cages were copolymerised with 1,4‐divinylbenzene (DVB) via radical polymerisation, giving amorphous Au@rCC3•HSS⊃PolyDVB and rCC3•HSS⊃PolyDVB with surface areas ranging from 410 to 836 m^2^ g^−1^), depending on the cage/DVB ratio employed during the polymerisation. Through a combination of scanning electron microscopy (SEM) and elemental mapping, a uniform distribution of the Au NPs with sizes around 0.67 nm was demonstrated. Au@rCC3•HSS⊃PolyDVB was employed for the catalytic degradation of cationic dyes such as methylene blue. In this way, the negatively charged surface of Au@rCC3•HSS⊃PolyDVB enhanced the catalytic degradation with a reaction constant of 0.1316 min^−1^, which is twenty times faster than for the non‐confined and cationic rCC3•HSS (0.00542 min^−1^), which was attributed to the electrostatic repulsion with the dye. Finally, the materials were evaluated for enzymatic catalysis, revealing that the Au@rCC3•HSS⊃PolyDVB accelerates the conversion of glucose to gluconic acid and *o*‐phenylenediamine to 2,3‐diaminophenazine at a rate ten times higher than that of its individual components. M. Liu, R. P. Lively, and co‐workers reported the dry‐jet wet‐quenching impregnation of cellulose acetate (CA) with CC3 by spinning, reaching a cage‐loading of around 60%.^[^ ^90^ ^]^ CC3 was shown to be stable after exposure to spinning solvents, as confirmed by NMR, PXRD, and gas sorption experiments, giving CC3⊃CA with enhanced porosity compared to the original polymer due to the intrinsic porosity of CC3. The addition of the POC during the spinning process did not affect the structural integrity of CA or CC3, whereas the pelletised CC3 counterparts suffered significant losses in textural properties. The final materials were tested toward post‐combustion flue gas and Xe/Kr separation, demonstrating excellent separation properties in short periods of time in both cases.

Given the interconnected pores of cages, CC3⊃polymers‐based materials have strong potential as hosts for achieving homogeneous Li⁺ transport, a crucial process in battery applications. Building on this concept, P. Cai, K. Li, Q. Zheng, and collaborators envisioned using the octahedral CC3 to encapsulate bis(trifluoromethane)sulfonimide (TFSI) anions, leading to the development of a solid‐state electrolyte (Figure 11b).^[^ ^83^ ^]^ Through the solid impregnation of polytetrafluoroethylene (PTFE) with crystalline CC3 by mechanical grinding and using a roller press, CC3⊃PolyTFE films were obtained, which were further treated with LiTFSI to furnish the desired electrolytes. Cross‐sectional SEM micrographs revealed a film thickness of around 66 µm. The crystalline structure of CC3 and the morphology of the crystals homogeneously distributed along the host polymer were demonstrated by PXRD and SEM analysis. The hybrid material was tested as a solid‐state electrolyte, offering a conductivity of 1.25 × 10^−4^ S cm^−1^ with no capacity loss and excellent cycling performance, unlike the starting materials. The formation of lithium dendrites was not observed, an essential factor in mitigating battery degradation.

Finally, several research teams studied the formation of Cage⊃Polymers mixed matrix membranes, produced by co‐dissolution of cages and polymers and subsequent drop‐casting for gas separation,^[^ ^91^, ^92^, ^93^ ^]^ showing increased permeability and selectivity with reference to the bare polymers.

## 5 Metal Organic Frameworks

MOFs are a class of hybrid inorganic‐organic frameworks produced by poly‐coordination of organic linkers with metal nodes, giving access to porous materials with long‐range order,^[^ ^94^, ^95^ ^]^ Most commonly, polydentate organic linkers bearing coordinating functional groups such as carboxylates or amines are used to form dative bonds with metal salts.^[^ ^96^ ^]^ The variation of metal cations and the organic linkers, also known as secondary building units (SBUs), can tailor the MOFs structure. Thus, in function of the symmetry of the SBU (linear, trigonal, tetrahedral) or the formation of different metal clusters, MOFs with different topologies or lattices can be obtained.^[^ ^97^ ^]^ In addition, the high directionality of coordination bonds and the high dynamism observed for some dative interactions, which favours the self‐assembly process,^[^ ^98^ ^]^ often lead to larger crystalline domains than those observed for COFs. Thanks to this reversibility, the formation process allows the usage of linkers with certain degrees of flexibility, unlike COFs, which usually require significant directionality for the covalent bond formation to achieve a consistent, rigid network. This effect is evident when comparing the number of reported chiral MOFs, typically obtained using chiral SBUs,^[^ ^95^ ^]^ with chiral COFs, which remain few in number.^[^ ^99^ ^]^ The employment of POCs as SBUs for the formation of new MOFs is largely unexplored. Due to the great number of coordinative moieties that are passively installed during the assembly of POCs (e.g., amines or imines)^[^ ^96^, ^100^ ^]^ as well as the functional groups that can be incorporated into the POC by orthogonal approaches or postmodifications,^[^ ^97^, ^101^ ^]^ cages can serve as a versatile tool to produce novel MOFs of unknown complexity. The intrinsic pores of the periodically arranged POCs can also be accessed, yielding a complex pore‐in‐pore network. Moreover, the coordinative bonds can be employed to arrange the POCs periodically in high‐quality crystals with narrow and pre‐designable pore size distributions featuring tunable nano‐environments, which is highly desirable in areas such as adsorption or separation. In addition, the resulting lattices could be designed a priori, a process that is now being revolutionised by AI‐assisted tools and deep learning.^[^ ^102^ ^]^ In addition, it is reported that the construction of Cage‐in‐MOFs can enhance the chemical stability of the frameworks,^[^ ^103^, ^104^ ^]^ presumably due to the stabilisation of the void spaces. Although imine‐linked cages result in frameworks that are less chemically stable than their amine‐linked counterparts, this methodology opens up opportunities for breakthrough applications in previously inaccessible fields. Similar to the Cage‐COFs, cage‐based linkers could open the way toward Cage‐MOFs with predictable partitioned cavities, enabling new levels of hierarchically organised porosity. Owing to the excellent processability and performance of molecular POCs in solution and applications like the study of host–guest interactions^[^ ^101^ ^]^ or anion recognition,^[^ ^105^ ^]^ the combinatorial platform of POCs and MOFs offers promising synergy to utilise their permanent porosity in the solid‐state. In addition, cage‐based linkers could succeed in certain applications where conventional SBUs fail, such as the translation of host–guest chemistry to the solid‐state,^[^ ^101^ ^]^ or the selective adsorption of guests.^[^ ^97^ ^]^

### 5.1 Sequential Synthesis of Cage‐MOFs

The first example of Cage‐MOFs using pre‐assembled cages was reported by A. I. Cooper and colleagues in 2010,^[^ ^96^ ^]^ with the amine‐functionalised rCC3 being used for the framework formation. rCC3‐Zn‐MOF was successfully obtained through coordination with zinc nitrate, resulting in a porous framework with long‐range order. rCC3‐Zn‐MOF crystallises in the space group *F23*, containing zinc carbonate clusters in the Wyckoff sites 4a, while the linking cages are occupying the sites 4b. Each cage exhibits an octahedral arrangement of Zn(II) cations interconnected by four carbonate anions in a *μ_3_*‐fashion. The coordination bond is formed between two amine groups on one edge of each rCC3 moiety and the Zn clusters. While the Zn clusters have a diameter of 8.5 Å, the POCs exhibit a larger size of 10.4 Å. The framework features large voids of 12 Å, formed during the crystallisation of the rCC3‐Zn‐MOF, which are filled with water and counter anions. The framework showed great thermal stability up to 330 °C under an inert atmosphere, and a phase transition was observed at 120 °C without the loss of crystallinity, demonstrating the feasibility of the Cage‐to‐MOF strategy to obtain stable and periodic cage‐based frameworks. rCC3‐Zn‐MOF was tested for CO_2_ adsorption, showing an uptake of 0.9 mmol g^−1^ (4 wt%) CO_2_ at 298 K and 1 bar.

**Figure 12:**
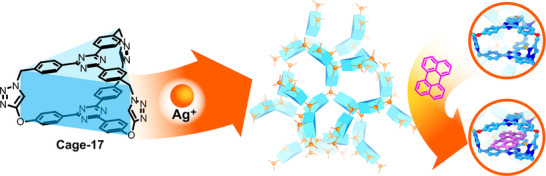
Synthesis of Cage‐17‐Ag‐MOF highlighting the sorption site of PAHs (pyrene). Cage‐17 is represented as blue trigonal prisms, and Ag cations as orange spheres.^[^ ^97^ ^]^

Based on this work, W. Huang, Y. Pan and colleagues explored the formation of Cage‐16‐Na‐MOF via imine coordination of a tube‐like Cage‐16, which resembles a double‐walled trianglimine macrocycle.^[^ ^100^ ^]^ The imine‐based barrel was further stabilised by the incorporation of hydroxyl groups, which act as proton donors for the intramolecular hydrogen bonding, dubbed imine‐clip formation.^[^ ^11^ ^]^ Cage‐16 was assembled into a helical crystalline structure via sodium coordination involving ─OH and ─N═ groups. These one‐dimensional helices are arranged into a three‐dimensional crystalline framework, stabilised by van der Waals interactions, and exhibit hierarchically organised porosity. The material showed a higher surface area than the pristine cage (1230 versus 881 m^2^ g^−1^), confirming that the networked cages show enhanced porosity. It was successfully employed as a CO_2_ adsorbent and separator, displaying high uptakes and selectivity, unlike the pristine POC. Following this principle, R. Natarajan and colleagues synthesised a 1,2,3‐triazole‐linked Cage‐17 featuring a trigonal‐prism shape and converted these into Cage‐17‐Ag‐MOF‐ by Ag(I) coordination (Figure 12).^[^ ^97^ ^]^ The resulting framework crystallised with an *srs* topology, exhibiting a non‐interpenetrated structure in the *P*2_1_2_1_2_1_ space group. The Ag(I) cations interconnected the cages in a planar trigonal configuration through the nitrogen atoms of the triazole rings, forming the extended framework. Interestingly, the compound was obtained as single‐crystals in an *M* and *P* racemic mixture, which could be selectively inducted using *R* or *S* isomers of camphor sulphonate during the network formation. The framework presents hierarchical porosity produced by the periodic rearrangement of the cages, and two channels can be distinguished. The material showed excellent stability toward a wide number of polar and non‐polar solvents and even against pH variations ranging from 3 to 10, retaining the diffraction pattern in PXRD measurements. The Cage‐17‐MOF showed low porosity values due to the small dimensions of the cages, exhibiting uptakes of 0.8 mmol g^−1^ of CO_2_ at 1 bar and 273 K, 87 cm^3^ g^−1^ at standard temperature and pressure (STP) of methanol, 69 cm^3^ g^−1^ STP of water, and 56 cm^3^ g^−1^ STP of toluene. Furthermore, the adsorption of a wide variety of PAHs was studied, where Cage‐17‐Ag‐MOF adsorbed up to 98% of the smaller carcinogens and up to 93% of the larger ones. This process was demonstrated even in water and at ppb concentrations, with material recyclability. Similar works were reported by K. Wen^[^ ^106^ ^]^ and colleagues or Z. Chen´s research team^[^ ^107^ ^]^ based on oxa‐cages showing the excellent structural versatility that can be incorporated into extended frameworks using POCs as SBUs.

**Figure 13:**
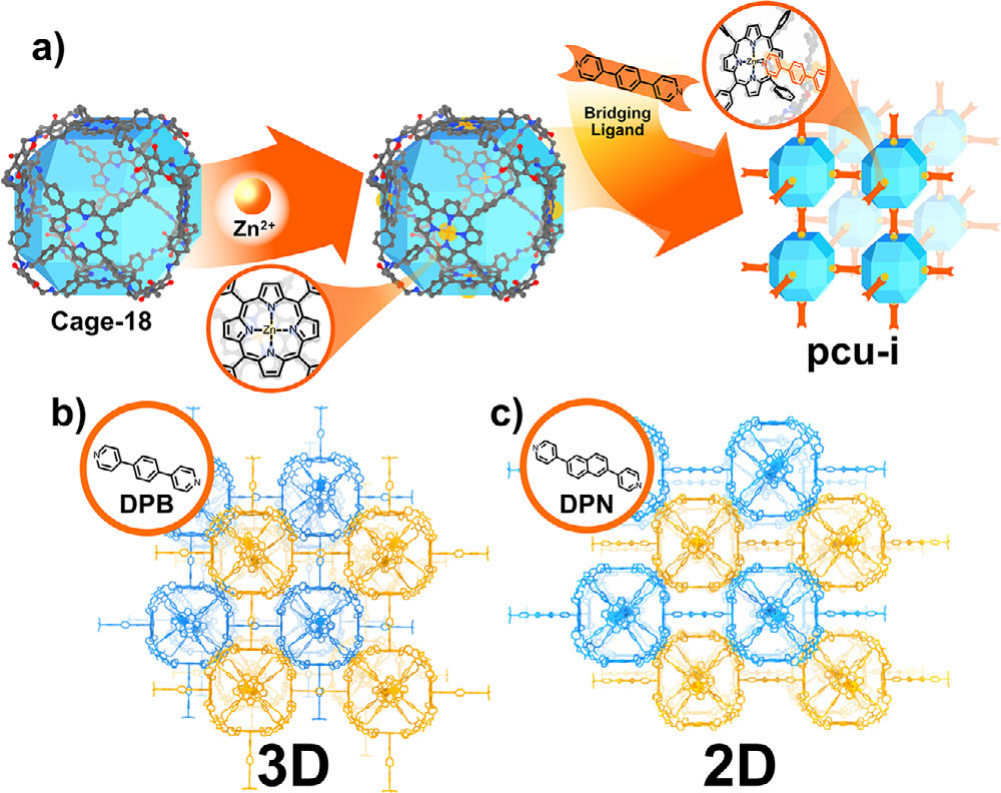
Cage‐MOF based on porphyrin boxes (blue polyhedron).^[^ ^108^ ^]^ a) Zn(II) chelation (orange spheres) and subsequent formation of a *pcu‐i* network by axial coordination (inlet‐depicted); b) 3D‐framework of Cage‐18‐DPE‐MOF highlighting the double‐interpenetrated structure; c) 2D‐framework of Cage‐18‐DPN‐MOF highlighting the AB‐layered structure.

K. Kim's research team reported the synthesis of a novel Cage‐MOF employing the porphyrin‐containing POC (Cage‐18) in 2018 (Figure 13).^[^ ^108^ ^]^ The six pyrrolic rings of each POC were metalated with Zn(II) cations to yield Zn‐Cage‐18. Then, the axial positions of the metallo‐POCs were coordinated with a bipyridine‐based ditopic linker, 1,4‐di(4‐pyridyl)benzene (DPB), affording a 3D Cage‐18‐DPB‐MOF, which crystallises in the *Pn‐3n* space group with a two‐fold interpenetration (Figure 13a). The framework displayed a substantial void volume provided intrinsically by Cage‐18 and an extrinsic pore. To test the generality of this approach, several ditopic linkers of varying lengths were tested: The shorter 1,2‐di(4‐pyridyl)ethylene (DPE) yielded a Cage‐18‐DPE‐MOF network with smaller unit cells. Strikingly, the employment of 2,6‐di(pyridine‐4‐yl) naphthalene (DPN) yielded Cage‐18‐DPN‐MOF with a 2D‐layer structure of AB‐stacked networks (Figure 13b). In this framework the metallo‐cage was only chelated through the four equatorial positions of the cuboid, instead of the six coordinative positions, which would have yielded a 3D superstructure, as demonstrated through PXRD experiments. The surface areas were analysed by measuring the respective N_2_ sorption isotherms at 77 K, revealing the increased porosity of all the Cage‐MOFs presented in comparison to the pure metallo‐Cage‐18 (539 m^2^ g^−1^), showing values of 1960, 1100, and 1090 m^2^ g^−1^ for Cage‐18‐DPB‐MOF, Cage‐18‐DPE‐MOF, and Cage‐18‐DPN‐MOF, respectively. As a proof of concept, the material was employed as a photocatalyst for the generation of singlet oxygen, revealing that Cage‐18‐DPB‐MOF exhibited enhanced reaction kinetics.

Related to the last work, W. Zhang's group designed a porphyrin cage with complementary size to fullerenes for solid‐state guest recognition.^[^ ^101^ ^]^ They synthesised the cubic Cage‐19 bearing two porphyrin faces and carbazole vertices through a one‐step dimerisation of the tetrapodal porphyrin precursor by alkyne metathesis in good overall yields. In addition, the carbazole vertices were decorated with carboxylic acids to produce the Cage‐19‐Zr‐MOF by coordination of ZrOCl_2_. The material was obtained as single‐crystals exceeding 100 µm in all dimensions and were further characterised by SC‐XRD revealing a tetragonal *P*4/*mmm* space group with panel‐to‐panel lengths of 14.3 Å. In addition, the crystals showed a high surface area of 742 m^2^ g^−1^ beneficial for guest recognition. Due to the large aromatic cavities, the Cage‐19‐Zr‐MOF was studied for selective adsorption of C_70_ over C_60_, showing affinity constants of 0.1161 and 0.06545 mg g^−1^ min^−1^, respectively. The authors also demonstrated the isolation of both compounds with breakthrough separation of a fullerene mixture with Cage‐19‐Zr‐MOF as an additive and reusability.

In 2022, Y. Chen and colleagues investigated the exfoliation process of 2D‐Cage‐MOFs. To achieve this, they synthesised a triazine‐triimidazole‐based Cage‐20, which serves as a tritopic linker with a trigonal prism‐like shape.^[^ ^109^ ^]^ The reaction of Cage‐20 with Zn and Cd salts yielded the Cage‐20‐Zn‐MOF and Cage‐20‐Cd‐MOF, respectively, both crystallising in the trigonal *R*3̅ space group. The structures were determined by SC‐XRD, revealing an AB‐layered configuration with inter‐network spacings around 4.5 Å pierced by triangular pores with sizes around 1 nm. The coordination remains in the 2D plane, while the crystal is sustained in the a‐c plane through intermolecular forces with origins in weak C─H⋯π interactions between the imidazole rings and the phloroglucinol moieties. The delamination process was studied by LPE assisted by ultrasound, and the Cage‐MOF nanosheets (MONs) were isolated by sedimentation and centrifugation. The nanolayers obtained were characterised by transmission electron microscopy (TEM) and atomic force microscopy (AFM), revealing the successful exfoliation of the Cage‐20‐Zn‐MOF. The MON's thickness was around 1.1 nm, consistent with a few‐layered material, and presented excellent evenness and homogeneity with lateral sizes up to 10 µm. With this work, the authors reported a straightforward method to obtain thin MONs with Cage‐20 as building blocks. It is worth emphasising that obtaining few‐layered materials via LPE is not trivial since it requires exquisite control of the external parameters (solvent, ultrasonic power, time, and temperature).^[^ ^110^, ^111^ ^]^ In this area of research, the use of cages as linkers in framework constructions is particularly intriguing, as they may enhance exfoliation feasibility by reducing interlayer interactions. Worth pointing out in this context are also the recent works of I. Imaz and D. Maspoch, which are essentially showcasing the opposite strategy with their clip‐off chemistry‐based approach, essentially carving out MOC subunits from the framework materials by ozonolysis.^[^ ^112^, ^113^, ^114^ ^]^

## 6 Supramolecular Frameworks

POCs can form supramolecular frameworks (sometimes called SOFs) through self‐assembly, with their polyhedral geometry particularly well‐suited for generating new porous structures.^[^ ^115^ ^]^ This classification encompasses cage‐based solids (crystalline or amorphous),^[^ ^12^, ^116^ ^]^ thin films,^[^ ^117^ ^]^ and hydrogen‐bonded organic frameworks (HOFs), which are a type of POC‐based crystal stabilised by hydrogen bonding.^[^ ^118^, ^119^, ^120^ ^]^ We focus on halogen‐bonded frameworks (HBFs) in this review, as they represent a common mode of self‐assembly found in organic solids. HBFs arise from the co‐assembly of a halogen bond donor, or Lewis acid (typically a highly polarised halogen such as Cl, Br, or I), and a halogen bond acceptor, or Lewis base (usually a highly electronegative atom such as N, O, or S). This interaction is highly directional and tuneable, allowing the formation of a wide variety of structures.^[^ ^121^ ^]^ In addition, if cooperative intermolecular interactions are present, for instance, π–π interactions by using aromatic building blocks, the stability of the framework is greatly enhanced.^[^ ^119^ ^]^ Since the packing of POCs significantly influences their properties, this strategy could serve as a useful tool for achieving permanent porosity.^[^ ^122^ ^]^

### 6.1 Sequential Synthesis of HBFs

**Figure 14:**
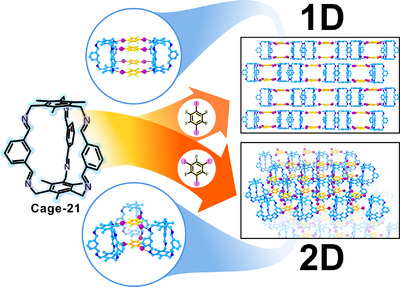
Synthesis of 1D‐ and 2D‐HBFs using Cage‐21. Top: halogen‐bonding with DITF; bottom: halogen‐bonding with TITF.^[^ ^123^ ^]^

O. Weingart, B. M. Schmidt, and colleagues reported the co‐assembly of Cage‐21 and Cage‐22 with 1,4‐diiodotetrafluorobenzene (DITF) and 1,3,5‐triiodotrifluorobenzene (TITF), yielding three different Cage‐HBFs using the imine bond for network formation (Figure 14).^[^ ^123^ ^]^ The halogen bonding was also studied in solution and by plane‐wave density functional theory (DFT) calculations and quantum theory of atoms in molecules (QTAIM) analyses of the entire unit cells, demonstrating that the imines can indeed form N⋯I─C halogen bonds with imines. The co‐assembly of Cage‐21 with DITF produced layered supramolecular structures, which grow along the *c* axis through π–π interactions, with inter‐cage distances of around 13 Å featuring a cubic‐like void space. The framework obtained by the co‐assembly with TITF produced smaller triangular‐prismatic cavities of sizes around 9 Å, showing the versatility of this approach for producing tailor‐made materials. Interestingly, the co‐assembly of Cage‐22 with DIFT and TITF produced 3D frameworks due to the relative disposition of the imine functionalities. The authors also demonstrated that the formation of the crystalline structures can be formed by mechanochemistry, reducing the amount of solvent required for the crystallisation.

Q. Yan and colleagues employed weak P═O⋯I─Ar halogen‐bonding interactions.^[^ ^115^ ^]^ By assembling phosphine‐functionalised trigonal‐prism‐like amine cage (Cage‐23) as a ditopic‐halogen‐bond acceptor with two different halogen bond donors (D2I and D4I), Cage‐HBFs were obtained. Using different numbers of iodine donors, these supramolecular structures extend in one or two dimensions, respectively, leading to Cage‐23‐D2I‐HBF threads or Cage‐23‐D4I‐HBF nanosheets. The threadlike material exhibited a one‐dimensional structure with a uniform diameter of 1.8 nm and microscale lengths, as confirmed by AFM and TEM. Notably, filament diameter closely matches the cage size, suggesting that the material is constructed from single‐stranded cages, supported by high‐angle annular dark‐field scanning TEM (HAADF‐STEM). PXRD confirmed the material's periodicity, with diffraction peaks matching intercage distances. Use of tetratopic D4I led to a two‐dimensional structure, and microscopy revealed square‐shaped nanosheets of Cage‐23‐D4I‐HBF. Furthermore, TEM analysis showed a periodic tetragonal lattice featuring nanopores of around 3 Å. The structural organisation was further confirmed by PXRD, which evidenced the long‐range order within the nanomaterials. The porosity of the nanomaterials was studied by N_2_ sorption isotherms at 77 K, revealing increased surface areas from 281 m^2^ g^−1^ (Cage‐23) to 317 m^2^ g^−1^ for Cage‐23‐D2I‐HBF threads and 654 m^2^ g^−1^ Cage‐23‐D4I‐HBF nanosheets. The materials were employed as catalysts, showing that the 2D nanosheets were able to catalyse cycloadditions.

## 7 Summary and Outlook

The rising role of POCs as versatile synthons for framework materials represents a transformative advancement for a diverse array of applications. Hybrid cage‐based frameworks offer remarkable tunability, structural precision, and unprecedented opportunities in emerging technologies. Translating classical solution‐phase phenomena, such as host–guest chemistry, into solid‐state architectures further amplifies their functionality, paving the way for next‐generation materials. Among these, Cage‐COFs emerge as an exciting, yet nascent, class of porous solids. The synergy between fully organic POCs and COFs yields exceptionally lightweight materials that combine structural robustness with exquisite control over hierarchical porosity. The largely unexplored host‐in‐host strategies, the use of cage‐based linkers, and untapped cage‐like COF topologies represent an intriguing frontier for future innovation. Similarly, Cage‐POPs have attracted significant attention due to their exceptional chemical and thermal stability, which is advantageous for applications in harsher environments. Notably, their successful fabrication into macroscopic forms such as membranes and freestanding films positions them strongly for industrial adoption. Incorporating POCs into POPs introduces predictability into the otherwise irregular porosity of polymeric networks, enabling tailor‐made materials for selective adsorption and efficient separation. The emerging class of Cage⊃Polymer hybrids further extends the versatility of POCs, offering novel polymeric materials endowed with distinctive structural and functional characteristics that conventional polymers alone cannot achieve. Such hybrids present promising platforms for advanced adsorption, separation, and transport applications, with future research likely to explore their structural complexity and responsiveness to external stimuli. The high reversibility of coordinative bonds allows the formation of complex lattices, particularly when combined with the multiple chelating sites that can be introduced on the periphery of POCs, opening new avenues for Cage‐MOF design. Moreover, this strategy offers a straightforward means of activating POC porosity, as these compounds often contain heteroatoms with lone electron pairs capable of forming dative bonds. POC‐based nodes may surpass conventional SBUs in applications such as guest adsorption, separation, and catalysis. Nonetheless, significant challenges remain, including the synthesis of MOFs with cage‐like cavities,^[^ ^103^, ^104^, ^124^, ^125^, ^126^ ^]^ Cage‐MOFs via host‐in‐host strategy^[^ ^127^ ^]^ and frameworks composed entirely of POC cavities. In contrast, the metal‐free HBFs are still at an early stage, despite significant advances in the synthesis and application of discrete POCs. POCs present ideal subunits to assemble HBFs since most of the dynamic covalent cages contain imine bonds or Lewis basic sites, with an available lone pair to take part in halogen bonding,^[^ ^123^ ^]^ which is also true for many covalent cages.^[^ ^32^, ^42^ ^]^ These frameworks present the advantage of recyclability since they can be formed and recovered, hypothetically. However, their lower stability might hinder their application. Looking ahead, integrating the distinct advantages of discrete POCs with ordered framework assemblies, polymer reinforcements, and supramolecular architectures is poised to revolutionise the field. The continued exploration of adaptive, recyclable, and high‐performance porous materials will undoubtedly unlock new potentials across catalysis, selective separations, and beyond. Looking forward, interdisciplinary research combining advanced computational modelling, innovative synthetic strategies, and responsive smart cage systems will enable the transition of these materials from laboratory breakthroughs to industrial realities.

## Conflict of Interests

The authors declare no conflict of interest.

## Data Availability

Data sharing is not applicable to this article as no new data were created or analyzed in this study.

## References

[1] L. Shang , F. Ye , M. Li , Y. Zhao , Chem. Soc. Rev. 2022, 51, 4075–4093. 35502858 10.1039/d1cs01025e

[2] M. Raynal , P. Ballester , A. Vidal‐Ferran , P. W. N. M. van Leeuwen , Chem. Soc. Rev. 2014, 43, 1734–1787. 24365792 10.1039/c3cs60037h

[3] F. Beuerle , B. Gole , Angew. Chem. Int. Ed. 2018, 57, 4850–4878. 10.1002/anie.201710190 29205727

[4] X. Guan , F. Chen , Q. Fang , S. Qiu , Chem. Soc. Rev. 2020, 49, 1357–1384. 32067000 10.1039/c9cs00911f

[5] A. Khobotov‐Bakishev , L. Hernández‐López , C. von Baeckmann , J. Albalad , A. Carné‐Sánchez , D. Maspoch , Adv. Sci. 2022, 9, 2104753. 10.1002/advs.202104753 PMC9008419 35119223

[6] D. Li , A. Yadav , H. Zhou , K. Roy , P. Thanasekaran , C. Lee , Global Chall. 2024, 8, 2300244. 10.1002/gch2.202300244 PMC10862192 38356684

[7] K. S. Song , P. W. Fritz , A. Coskun , Chem. Soc. Rev. 2022, 51, 9831–9852. 36374129 10.1039/d2cs00727d PMC9703447

[8] Z. Xu , Y. Ye , Y. Liu , H. Liu , S. Jiang , Chem. Commun. 2024, 60, 2261–2282. 10.1039/d3cc05091b 38318641

[9] S. Ivanova , F. Beuerle , Isr. J. Chem. 2024, 64, e202400025.

[10] T. Kunde , T. Pausch , B. M. Schmidt , Eur. J. Org. Chem. 2021, 2021, 5844–5856.

[11] K. Acharyya , P. S. Mukherjee , Angew. Chem. Int. Ed. 2019, 58, 8640–8653. 10.1002/anie.201900163 30725512

[12] M. Mastalerz , Acc. Chem. Res. 2018, 51, 2411–2422. 30203648 10.1021/acs.accounts.8b00298

[13] A. I. Cooper , ACS Cent. Sci. 2017, 3, 544–553. 28691065 10.1021/acscentsci.7b00146 PMC5492258

[14] T. D. Bennett , F.‐X. Coudert , S. L. James , A. I. Cooper , Nat. Mater. 2021, 20, 1179–1187. 33859380 10.1038/s41563-021-00957-w

[15] S. Das , P. Heasman , T. Ben , S. Qiu , Chem. Rev. 2017, 117, 1515–1563. 28035812 10.1021/acs.chemrev.6b00439

[16] R. Freund , O. Zaremba , G. Arnauts , R. Ameloot , G. Skorupskii , M. Dincă , A. Bavykina , J. Gascon , A. Ejsmont , J. Goscianska , M. Kalmutzki , U. Lächelt , E. Ploetz , C. S. Diercks , S. Wuttke , Angew. Chem. Int. Ed. 2021, 60, 23975–24001. 10.1002/anie.202106259 33989445

[17] F. Chen , H. Zheng , Y. Yusran , H. Li , S. Qiu , Q. Fang , Chem. Soc. Rev. 2025, 54, 484–514. 39585733 10.1039/d4cs00703d

[18] T. Prieto , C. Ponte , R. Guntermann , D. D. Medina , L. M. Salonen , Chem.Eur. J. 2024, 30, e202401344. 38771916 10.1002/chem.202401344

[19] X. Chen , K. Geng , R. Liu , K. T. Tan , Y. Gong , Z. Li , S. Tao , Q. Jiang , D. Jiang , Angew. Chem. Int. Ed. 2020, 59, 5050–5091. 10.1002/anie.201904291 31144373

[20] T. Tateishi , M. Yoshimura , S. Tokuda , F. Matsuda , D. Fujita , S. Furukawa , Coord. Chem. Rev. 2022, 467, 214612.

[21] B. D. Egleston , A. Mroz , K. E. Jelfs , R. L. Greenaway , Chem. Sci. 2022, 13, 5042–5054. 35655552 10.1039/d2sc00087c PMC9093153

[22] T. Chen , Y. Li , Y. Wei , Y. Zhang , J. Zhu , B. Van der Bruggen , Sep. Purif. Technol. 2024, 330, 125440.

[23] X. Liu , P. Liu , H. Wang , N. M. Khashab , Adv. Mater. 2025, 37, 2500310. 10.1002/adma.202500310 40275732

[24] J.‐Y. Li , X.‐D. Yang , F.‐X. Chen , J.‐K. Sun , Mater. Chem. Front. 2023, 7, 5355–5376.

[25] E. Sánchez‐González , M. Y. Tsang , J. Troyano , G. A. Craig , S. Furukawa , Chem. Soc. Rev. 2022, 51, 4876–4889. 35441616 10.1039/d1cs00759a

[26] B. Lee , I.‐H. Park , J. Park , ACS Mater Lett. 2022, 4, 2388–2393.

[27] C. S. Diercks , O. M. Yaghi , Science 2017, 355, eaal1585. 28254887 10.1126/science.aal1585

[28] I. Irie , S. Das , Q. Fang , Y. Negishi , J. Am. Chem. Soc. 2025, 147, 1367–1380. 39745262 10.1021/jacs.4c14458

[29] K. Geng , T. He , R. Liu , S. Dalapati , K. T. Tan , Z. Li , S. Tao , Y. Gong , Q. Jiang , D. Jiang , Chem. Rev. 2020, 120, 8814–8933. 31967791 10.1021/acs.chemrev.9b00550

[30] X. Wu , X. Han , Y. Liu , Y. Liu , Y. Cui , J. Am. Chem. Soc. 2018, 140, 16124–16133. 30392376 10.1021/jacs.8b08452

[31] M. Jiménez‐Duro , E. Martínez‐Periñán , M. Martínez‐Fernández , J. I. Martínez , E. Lorenzo , J. L. Segura , Small 2024, 20, 2402082. 10.1002/smll.202402082 38773891

[32] J.‐X. Ma , J. Li , Y.‐F. Chen , R. Ning , Y.‐F. Ao , J.‐M. Liu , J. Sun , D.‐X. Wang , Q.‐Q. Wang , J. Am. Chem. Soc. 2019, 141, 3843–3848. 30773007 10.1021/jacs.9b00665

[33] Q. Zhu , X. Wang , R. Clowes , P. Cui , L. Chen , M. A. Little , A. I. Cooper , J. Am. Chem. Soc. 2020, 142, 16842–16848. 32893623 10.1021/jacs.0c07732 PMC7586335

[34] M. Li , Y. Peng , F. Yan , C. Li , Y. He , Y. Lou , D. Ma , Y. Li , Z. Shi , S. Feng , New J. Chem. 2021, 45, 3343–3348.

[35] M. J. Kory , M. Wörle , T. Weber , P. Payamyar , S. W. van de Poll , J. Dshemuchadse , N. Trapp , A. D. Schlüter , Nat. Chem. 2014, 6, 779–784. 25143212 10.1038/nchem.2007

[36] Z. Kahveci , T. Islamoglu , G. A. Shar , R. Ding , H. M. El‐Kaderi , CrystEngComm 2013, 15, 1524–1527.

[37] K. Cheng , H. Li , Z. Li , P.‐Z. Li , Y. Zhao , ACS Materials Lett. 2023, 5, 1546–1555.

[38] Y. Xue , Q. Lin , X. Sun , D. Li , Y. Fu , Z. Li , Y. Shi , C. Luo , X. Gui , K. Xu , Small 2025, 21, 2501988. 10.1002/smll.202501988 40237118

[39] K. Cheng , H. Li , J.‐R. Wang , P.‐Z. Li , Y. Zhao , Small 2023, 19, 2301998. 10.1002/smll.202301998 37162443

[40] M. Li , J. Ma , B. Pan , J. Wang , ACS Appl. Mater. Interfaces 2022, 14, 57180–57188. 36516002 10.1021/acsami.2c17878

[41] Y.‐X. Ma , Z.‐J. Li , L. Wei , S.‐Y. Ding , Y.‐B. Zhang , W. Wang , J. Am. Chem. Soc. 2017, 139, 4995–4998. 28347136 10.1021/jacs.7b01097

[42] C. Ji , K. Su , W. Wang , J. Chang , E.‐S. M. El‐Sayed , L. Zhang , D. Yuan , CCS Chemistry 2022, 4, 3095–3105.

[43] H. Hang , Z. Wang , S. Liu , W. Li , S. Wei , M. Wang , L. Zhang , W. Lyu , S. Liu , X. Lu , Sep. Purif. Technol. 2025, 356, 129897.

[44] P. Bhandari , P. S. Mukherjee , ACS Catal. 2023, 13, 6126–6143.

[45] A. Giri , G. Shreeraj , T. K. Dutta , A. Patra , Angew. Chem. Int. Ed. 2023, 62, e202219083. 10.1002/anie.202219083 36912437

[46] Z. Shan , X. Wu , B. Xu , Y.‐L. Hong , M. Wu , Y. Wang , Y. Nishiyama , J. Zhu , S. Horike , S. Kitagawa , G. Zhang , J. Am. Chem. Soc. 2020, 142, 21279–21284. 33295765 10.1021/jacs.0c11073

[47] G. Shreeraj , M. Tiwari , V. R. Dugyala , A. Patra , Langmuir 2024, 40, 16419–16429. 39042836 10.1021/acs.langmuir.4c01709

[48] Q. Song , J. Yang , K. Zheng , T. Zhang , C. Yuan , L.‐M. Yuan , X. Hou , J. Am. Chem. Soc. 2024, 146, 7594–7604. 38462726 10.1021/jacs.3c13692

[49] X. Kang , X. Han , C. Yuan , C. Cheng , Y. Liu , Y. Cui , J. Am. Chem. Soc. 2020, 142, 16346–16356. 32841013 10.1021/jacs.0c06605

[50] C. Gropp , T. Ma , N. Hanikel , O. M. Yaghi , Science 2020, 370, eabd6406. 33093081 10.1126/science.abd6406

[51] C. Qian , X. Zhao , Acc. Chem. Res. 2025, 58, 1192–1209. 40070122 10.1021/acs.accounts.4c00799

[52] H. M. El‐Kaderi , J. R. Hunt , J. L. Mendoza‐Cortés , A. P. Côté , R. E. Taylor , M. O'Keeffe , O. M. Yaghi , Science 2007, 316, 268–272. 17431178 10.1126/science.1139915

[53] J. R. Hunt , C. J. Doonan , J. D. LeVangie , A. P. Côté , O. M. Yaghi , J. Am. Chem. Soc. 2008, 130, 11872–11873. 18707184 10.1021/ja805064f

[54] “RCSR” , http://rcsr.net/layers (accessed: April 2025).

[55] S. Das , H. Mabuchi , T. Irie , K. Sasaki , M. Nozaki , R. Tomioka , D. Wen , Y. Zhao , T. Ben , Y. Negishi , Small 2024, 20, 2307666. 10.1002/smll.202307666 38279566

[56] Z. Sun , P. Li , S. Xu , Z.‐Y. Li , Y. Nomura , Z. Li , X. Liu , S. Zhang , J. Am. Chem. Soc. 2020, 142, 10833–10840. 32433875 10.1021/jacs.0c03330

[57] T. Prakasam , S. K. Sharma , F. Ravaux , F. Benyettou , M. Lusi , V. Sabu , P. Bazin , T. Delclos , R. Jagannathan , J. Whelan , M. El‐Roz , M. A. Olson , M. Abdellatief , O. S. Mudraj , F. Gándara , A. Trabolsi , Chem 2025, 11, 102307.

[58] X. Yang , Z. Ullah , J. F. Stoddart , C. T. Yavuz , Chem. Rev. 2023, 123, 4602–4634. 37023354 10.1021/acs.chemrev.2c00667 PMC10141292

[59] Z. Wang , H. Ma , T.‐L. Zhai , G. Cheng , Q. Xu , J.‐M. Liu , J. Yang , Q.‐M. Zhang , Q.‐P. Zhang , Y.‐S. Zheng , B. Tan , C. Zhang , Adv. Sci. 2018, 5, 1800141. 10.1002/advs.201800141 PMC6051374 30027046

[60] F. Begar , M. Erdogmus , Y. Gecalp , U. C. Canakci , O. Buyukcakir , ACS Appl. Polym. Mater 2024, 6, 5358–5365.

[61] T. Ashirov , J. Lim , A. Robles , T. Puangsamlee , P. W. Fritz , A. Crochet , X. Wang , C. Hewson , P. Iacomi , O. Š. Miljanić , A. Coskun , Angew. Chem. Int. Ed. 2025, 64, e202423809. 10.1002/anie.202423809 39804699

[62] Y. Jin , B. A. Voss , R. McCaffrey , C. T. Baggett , R. D. Noble , W. Zhang , Chem. Sci. 2012, 3, 874–877.

[63] Y. Jin , B. A. Voss , A. Jin , H. Long , R. D. Noble , W. Zhang , J. Am. Chem. Soc. 2011, 133, 6650–6658. 21473590 10.1021/ja110846c

[64] W. Lin , L. Cao , X. Liu , L. O. Alimi , J. Wang , B. A. Moosa , Z. Lai , N. M. Khashab , J. Am. Chem. Soc. 2024, 146, 34528–34535. 39533477 10.1021/jacs.4c11709

[65] A. Khobotov‐Bakishev , P. Samanta , K. Roztocki , J. Albalad , S. Royuela , S. Furukawa , F. Zamora , A. Carné‐Sánchez , D. Maspoch , Adv. Funct. Mater. 2024, 34, 2312166.

[66] X. Li , W. Lin , V. Sharma , R. Gorecki , M. Ghosh , B. A. Moosa , S. Aristizabal , S. Hong , N. M. Khashab , S. P. Nunes , Nat. Commun. 2023, 14, 3112. 37253741 10.1038/s41467-023-38728-7 PMC10229579

[67] A. Zhao , M. Zhang , Y. Bao , L. Zhao , G. Liu , Y. Jiang , P. Zhang , X. Cao , J. Membr. Sci. 2022, 664, 121081.

[68] O. Buyukcakir , Y. Seo , A. Coskun , Chem. Mater. 2015, 27, 4149–4155.

[69] Z. Wang , Y.‐Q. Liu , Y.‐H. Zhao , Q.‐P. Zhang , Y.‐L. Sun , B.‐B. Yang , J.‐H. Bu , C. Zhang , RSC Adv. 2022, 12, 16486–16490. 35754863 10.1039/d2ra02343a PMC9168829

[70] Z. Wang , Q. Ou , H. Ma , G. Cheng , Q.‐P. Zhang , B. Tan , C. Zhang , ACS Appl. Polym. Mater 2021, 3, 171–177.

[71] E. Martínez‐Ahumada , D. He , V. Berryman , A. López‐Olvera , M. Hernandez , V. Jancik , V. Martis , M. A. Vera , E. Lima , D. J. Parker , A. I. Cooper , I. A. Ibarra , M. Liu , Angew. Chem. Int. Ed. 2021, 60, 17556–17563. 10.1002/anie.202104555 PMC8361948 33979473

[72] T. Tozawa , J. T. A. Jones , S. I. Swamy , S. Jiang , D. J. Adams , S. Shakespeare , R. Clowes , D. Bradshaw , T. Hasell , S. Y. Chong , C. Tang , S. Thompson , J. Parker , A. Trewin , J. Bacsa , A. M. Z. Slawin , A. Steiner , A. I. Cooper , Nat. Mater. 2009, 8, 973–978. 19855385 10.1038/nmat2545

[73] M. Liu , M. A. Little , K. E. Jelfs , J. T. A. Jones , M. Schmidtmann , S. Y. Chong , T. Hasell , A. I. Cooper , J. Am. Chem. Soc. 2014, 136, 7583–7586. 24785267 10.1021/ja503223j

[74] T. Pausch , T. David , T. Fleck‐Kunde , H. Pols , J. Gurke , B. M. Schmidt , Angew. Chem. Int. Ed. 2024, 63, e202318362. 10.1002/anie.202318362 38294139

[75] M. Jiménez‐Duro , R. Barranco‐García , M. Martínez‐Fernández , B. Asenjo‐Filgueira , J. I. Martínez , R. Cuervo‐Rodríguez , A. Muñoz‐Bonilla , M. Fernández‐García , J. L. Segura , ACS Appl. Mater. Interfaces 2024, 16, 70883–70890. 39670463 10.1021/acsami.4c16938

[76] S. Bhattacharya , R. S. Phatake , S. Nabha Barnea , N. Zerby , J.‐J. Zhu , R. Shikler , N. G. Lemcoff , R. Jelinek , ACS Nano 2019, 13, 1433–42. 30615415 10.1021/acsnano.8b07087

[77] M. Martínez‐Fernández , R. Gavara , S. Royuela , L. Fernández‐Ecija , J. I. Martínez , F. Zamora , J. L. Segura , J. Mater. Chem. A 2022, 10, 4634–4643.

[78] G. Romo‐Islas , R. Gavara , Inorganics 2021, 9, 32.

[79] A. Chiloeches , A. Funes , R. Cuervo‐Rodríguez , F. López‐Fabal , M. Fernández‐García , C. Echeverría , A. Muñoz‐Bonilla , Polym. Chem. 2021, 12, 3190–3200.

[80] T. Uemura , R. Nakanishi , S. Mochizuki , S. Kitagawa , M. Mizuno , Angew. Chem. Int. Ed. 2016, 55, 6443–6447. 10.1002/anie.201601587 27027409

[81] L. O. Alimi , F. Fang , B. Moosa , Y. Ding , N. M. Khashab , Angew. Chem. Int. Ed. 2022, 61, e202212596. 10.1002/anie.202212596 36047488

[82] J. Dechnik , J. Gascon , C. J. Doonan , C. Janiak , C. J. Sumby , Angew. Chem. Int. Ed. 2017, 56, 9292–9310. 10.1002/anie.201701109 28378379

[83] W.‐M. Qin , Z. Li , W.‐X. Su , J.‐M. Hu , H. Zou , Z. Wu , Z. Ruan , Y.‐P. Cai , K. Li , Q. Zheng , Nano‐Micro Lett. 2025, 17, 38. 10.1007/s40820-024-01499-x PMC11480285 39404929

[84] L. Tan , J.‐H. Zhou , J.‐K. Sun , J. Yuan , Nat. Commun. 2022, 13, 1471. 35304468 10.1038/s41467-022-29031-y PMC8933400

[85] T. M. del Campo , D. San Martín , L. Gamarra , E. Cerrón , S. Cembellín , H. Yanai , P. Almendros , J. Org. Chem. 2024, 89, 14228–14232. 39288304 10.1021/acs.joc.4c01648 PMC11460725

[86] T. Fernandes , R. Rani Mohan , L. Donk , W. Chen , C. Biz , M. Fianchini , S. Kamali , S. Mohammad Alizadeh , A. Kitayev , A. Ashdot , M. Page , L. M. Salonen , S. Kopp , E. Tal Gutelmacher , J. Gracia , M. Costa Figueiredo , Y. V. Kolen'ko , Energy Adv 2024, 3, 2575–2586.

[87] P. Wang , C. Gao , Q. Dong , L. Wang , D. Chu , Y. He , W. Bai , Colloids Surf. A: Physicochem. Eng. Asp. 2025, 705, 135654.

[88] P. Rani , R. Das , C. M. Nagaraja , Inorg. Chem. Front. 2025, 12, 430–478.

[89] J. S. Choi , G. V. Fortunato , M. Malinovic , E. S. Koh , R. Aymerich‐Armengol , C. Scheu , H. Wang , A. Hutzler , J. P. Hofmann , M. R. V. Lanza , M. Ledendecker , Nano Energy 2025, 137, 110811.

[90] I. Borne , D. He , S. J. A. DeWitt , M. Liu , A. I. Cooper , C. W. Jones , R. P. Lively , ACS Appl. Mater. Interfaces 2021, 13, 47118–47126. 34570486 10.1021/acsami.1c12002

[91] A. F. Bushell , P. M. Budd , M. P. Attfield , J. T. A. Jones , T. Hasell , A. I. Cooper , P. Bernardo , F. Bazzarelli , G. Clarizia , J. C. Jansen , Angew. Chem. Int. Ed. 2013, 52, 1253–1256. 10.1002/anie.201206339 PMC3734621 23225333

[92] G. Zhu , F. Zhang , M. P. Rivera , X. Hu , G. Zhang , C. W. Jones , R. P. Lively , Angew. Chem. Int. Ed. 2019, 58, 2638–2643. 10.1002/anie.201811341 30577090

[93] Q. Zhang , H. Li , S. Chen , J. Duan , W. Jin , J. Membr. Sci. 2020, 611, 118288.

[94] K. R. Ansari , A. Singh , M. Younas , I. H. Ali , Y. Lin , Coord. Chem. Rev. 2025, 523, 216294.

[95] W. Gong , Z. Chen , J. Dong , Y. Liu , Y. Cui , Chem. Rev. 2022, 122, 9078–9144. 35344663 10.1021/acs.chemrev.1c00740

[96] S. I. Swamy , J. Bacsa , J. T. A. Jones , K. C. Stylianou , A. Steiner , L. K. Ritchie , T. Hasell , J. A. Gould , A. Laybourn , Y. Z. Khimyak , D. J. Adams , M. J. Rosseinsky , A. I. Cooper , J. Am. Chem. Soc. 2010, 132, 12773–12775. 20795723 10.1021/ja104083y

[97] B. Paul , S. Ghorai , J. Samanta , R. Natarajan , Small 2025, 21, 2408482. 10.1002/smll.202408482 39618012

[98] H.‐C. “Joe” Zhou , S. Kitagawa , Chem. Soc. Rev. 2014, 43, 5415–5418. 25011480 10.1039/c4cs90059f

[99] X. Kang , E. R. Stephens , B. M. Spector‐Watts , Z. Li , Y. Liu , L. Liu , Y. Cui , Chem. Sci. 2022, 13, 9811–9832. 36199638 10.1039/d2sc02436e PMC9431510

[100] L. Zhang , L. Xiang , C. Hang , W. Liu , W. Huang , Y. Pan , Angew. Chem. Int. Ed. 2017, 56, 7787–7791. 10.1002/anie.201702399 28504831

[101] S. Huang , S. J. Teat , L. J. Wayment , N. S. Settineri , H. Chen , Z. Lei , W. Zhang , Angew. Chem. Int. Ed. 2024, 63, e202409432. 10.1002/anie.202409432 38946171

[102] Y. Zhao , Y. Zhao , J. Wang , Z. Wang , Ind. Eng. Chem. Res. 2025, 64, 4637–4668.

[103] D. Tian , Q. Chen , Y. Li , Y.‐H. Zhang , Z. Chang , X.‐H. Bu , Angew. Chem. Int. Ed. 2014, 53, 837–841. 10.1002/anie.201307681 24282117

[104] J.‐H. Liu , L.‐D. Lin , X.‐X. Li , D. Zhao , Y.‐Q. Sun , S.‐T. Zheng , Chem. Commun. 2019, 55, 7394–7397. 10.1039/c9cc03288f 31179455

[105] B. Qiao , J. R. Anderson , M. Pink , A. H. Flood , Chem. Commun. 2016, 52, 8683–8686. 10.1039/c6cc03463b 27331606

[106] W.‐J. Hu , L.‐Q. Liu , M.‐L. Ma , X.‐L. Zhao , Y. A. Liu , X.‐Q. Mi , B. Jiang , K. Wen , Inorg. Chem. 2013, 52, 9309–9319. 23927581 10.1021/ic400751n

[107] L. Shi , Z. Xiong , H. Wang , H. Cao , Z. Chen , Chem 2024, 10, 2464–2472.

[108] Y. Kim , J. Koo , I.‐C. Hwang , R. D. Mukhopadhyay , S. Hong , J. Yoo , A. A. Dar , I. Kim , D. Moon , T. J. Shin , Y. H. Ko , K. Kim , J. Am. Chem. Soc. 2018, 140, 14547–14551. 30272449 10.1021/jacs.8b08030

[109] P. Yang , J. Jiang , J.‐P. Ma , B. Zheng , Y. Yan , J. Wang , Y. Zou , Q. Liu , Y. Chen , Inorg. Chem. 2022, 61, 1521–1529. 34985269 10.1021/acs.inorgchem.1c03239

[110] D. Rodríguez‐San‐Miguel , C. Montoro , F. Zamora , Chem. Soc. Rev. 2020, 49, 2291–2302. 32182308 10.1039/c9cs00890j

[111] Y.‐Y. Xu , H. Cao , Y. Xue , B. Li , W. Cai , Nanomaterials 2018, 8, 942. 30445778 10.3390/nano8110942 PMC6265730

[112] D. Nam , J. Albalad , R. Sánchez‐Naya , S. Ruiz‐Relaño , A. Cortés‐Martínez , Y. Yang , J. Juanhuix , I. Imaz , D. Maspoch , J. Am. Chem. Soc. 2024, 146, 27255–27261. 39348446 10.1021/jacs.4c09077 PMC11468772

[113] S. Ruiz‐Relaño , D. Nam , J. Albalad , A. Cortés‐Martínez , J. Juanhuix , I. Imaz , D. Maspoch , J. Am. Chem. Soc. 2024, 146, 26603–26608. 39311525 10.1021/jacs.4c09431 PMC11450890

[114] A. Broto‐Ribas , S. Ruiz‐Relaño , J. Albalad , Y. Yang , F. Gándara , J. Juanhuix , I. Imaz , D. Maspoch , Angew. Chem. Int. Ed. 2023, 62, e202310354. 10.1002/anie.202310354 37671919

[115] Y. Wang , G.‐F. Mu , K.‐S. Sun , N. Yang , Q. Yan , ACS Mater. Lett. 2024, 6, 3667–3674.

[116] Y. Jiang , L. Wang , T. Yan , J. Hu , W. Sun , R. Krishna , D. Wang , Z. Gu , D. Liu , X. Cui , H. Xing , Y. Zhang , Angew. Chem. Int. Ed. 2013, 52, 3746–3749.

[117] A. He , Z. Jiang , Y. Wu , H. Hussain , J. Rawle , M. E. Briggs , M. A. Little , A. G. Livingston , A. I. Cooper , Nat. Mater. 2022, 21, 463–470. 35013552 10.1038/s41563-021-01168-z PMC8971131

[118] W. Wu , D. Chen , M. Zhang , X. Zhao , R. Zhao , C. Geng , J. Jia , G. Zhu , J. Am. Chem. Soc. 2025, 147, 2228–2236. 39754291 10.1021/jacs.4c17520

[119] B. Han , H. Wang , C. Wang , H. Wu , W. Zhou , B. Chen , J. Jiang , J. Am. Chem. Soc. 2019, 141, 8737–8740. 31117661 10.1021/jacs.9b03766 PMC7928070

[120] Q. Zhu , L. Wei , C. Zhao , H. Qu , B. Liu , T. Fellowes , S. Yang , A. Longcake , M. J. Hall , M. R. Probert , Y. Zhao , A. I. Cooper , M. A. Little , J. Am. Chem. Soc. 2023, 145, 23352–23360. 37824718 10.1021/jacs.3c09246 PMC10603795

[121] V. Nemec , K. Lisac , N. Bedeković , L. Fotović , V. Stilinović , D. Cinčić , CrystEngComm 2021, 23, 3063–3083.

[122] T. Hasell , J. L. Culshaw , S. Y. Chong , M. Schmidtmann , M. A. Little , K. E. Jelfs , E. O. Pyzer‐Knapp , H. Shepherd , D. J. Adams , G. M. Day , A. I. Cooper , J. Am. Chem. Soc. 2014, 136, 1438–1448. 24410310 10.1021/ja409594s

[123] E. Nieland , D. Komisarek , S. Hohloch , K. Wurst , V. Vasylyeva , O. Weingart , B. M. Schmidt , Chem. Commun. 2022, 58, 5233–5236. 10.1039/d2cc00799a 35388831

[124] W. Ji , G. Wang , B. Wang , B. Yan , L. Liu , L. Xu , T. Ma , S. Yao , Y. Fu , L. Zhang , Q. Zhai , Chinese J. Struct. Chem. 2023, 42, 100062.

[125] Y.‐P. Li , J.‐J. Ni , S.‐C. Fan , Q.‐G. Zhai , Chinese J. Struct. Chem. 2023, 42, 100070.

[126] Y. Jiang , L. Wang , T. Yan , J. Hu , W. Sun , R. Krishna , D. Wang , Z. Gu , D. Liu , X. Cui , H. Xing , Y. Zhang , Chem. Sci. 2023, 14, 298–309. 36687342 10.1039/d2sc05742e PMC9811657

[127] Q. Wu , J. Liang , D. Wang , R. Wang , C. Janiak , Chem. Soc. Rev. 2025, 54, 601–622. 39589788 10.1039/d4cs00371c

